# Monitoring Aerobic Marine Bacterial Biofilms on Gold Electrode Surfaces and the Influence of Nitric Oxide Attachment Control

**DOI:** 10.1021/acs.analchem.2c00934

**Published:** 2022-08-31

**Authors:** Stephane Werwinski, Julian A. Wharton, Mengyan Nie, Keith R. Stokes

**Affiliations:** † National Centre for Advanced Tribology at Southampton (nCATS), Faculty of Engineering and Physical Sciences, University of Southampton, Highfield, Southampton SO17 1BJ, U.K.; ‡ UCL Institute for Materials Discovery, University College London, Malet Place, London WC1E 7JE, U.K.; § Physical Sciences Department, Dstl, Porton Down, Salisbury, Wiltshire SP4 0JQ, U.K.

## Abstract

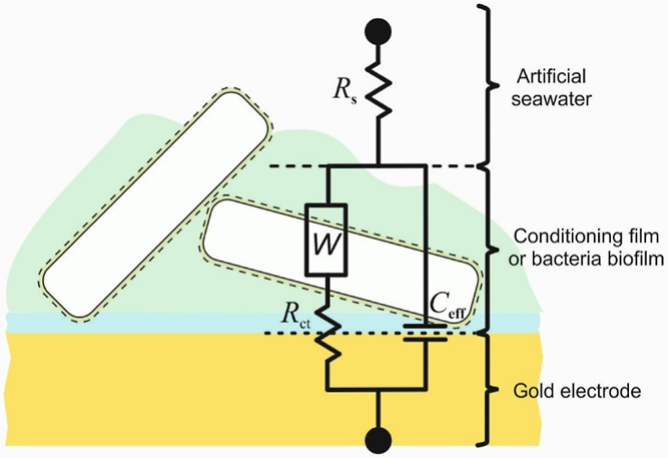

Detection of aerobic marine bacterial biofilms using electrochemical impedance spectroscopy has been done to monitor the interfacial response of *Pseudoalteromonas sp*. NCIMB 2021 attachment and growth in order to identify characteristic events on a 0.2 mm diameter gold electrode surface. Uniquely, the applicability of surface charge density has been proven to be valuable in determining biofilm attachment and cell enumeration over a 72 h duration on a gold surface within a modified continuous culture flow cell (a controlled low laminar flow regime with Reynolds number ≈ 1). In addition, biofilm dispersal has been evaluated using 500 nM sodium nitroprusside, a nitric oxide donor (nitric oxide is important for the regulation of several diverse biological processes). Ex situ confocal microscopy studies have been performed to confirm biofilm coverage and morphology, plus the determination and quantification of the nitric oxide biofilm dispersal effects. Overall, the capability of the sensor to electrochemically detect the presence of initial bacterial biofilm formation and extent has been established and shown to have potential for real-time biofilm monitoring.

## Introduction

Marine biofilms alter the hydrodynamic properties (surface frictional resistance can cause flow restrictions) and reduce the heat-transfer performance of operating marine heat exchangers, thus leading to failure and/or blockages.^1,2^ Biofilms are structured sessile microbial communities encapsulated within self-produced extracellular polymeric substances (EPSs) that adhere to wetted surfaces. Biofilm electrochemical sensors exploit the biofilm–electrode interface as the sensing element,^3−5^ where the biofilm electrochemical activity provides the principal sensing strategy, akin to a permeable biological membrane. In addition, enzymes such as catalase influence the oxygen reduction reaction (ORR); however, the exact interfacial mechanism attributed to aerobic biofilm action toward enzymatic processes on metallic surfaces needs elucidation.^6^ Common marine biofilm mitigation strategies use biocides; however, these are not always viable or can be ineffective, inefficient, and costly since dead microbes can be a substantial biomass for any pioneering bacterial attachment and growth.^1^ In addition, the increased ecological concerns and legislature have resulted in the restriction on the use of biocidal products.^1,2^ Overall, sensing surfaces are necessary, together with early warning systems that can quantitatively evaluate the metallic/seawater interface and inform on the extent of biofouling to determine a suitable and effective biocide dosing strategy. Biofilms undergo programed detachment and coordinated dispersal, where cells are released from mature biofilms for recolonization at other locations. Detachment was linked to the accumulation of reactive nitrogen species within biofilms.^7,8^ Physiological signaling molecules such as nitric oxide (NO), a biologically ubiquitous free-radical gas molecule, regulate biofilm dispersal.^7,9^ Exogenous exposure to low, nontoxic NO concentrations (nanomolar range, 500 nM) can induce dispersal in *Pseudomonas aeruginosa* biofilms and increase cell sensitivity to antimicrobial treatments. The NO concentration that induces dispersal is substantially below the concentration that would be toxic to biofilms.^7,8^ NO signaling thus exhibits a low-dose, economically attractive, and environmentally benign means for the control of biofouling. At concentrations that trigger dispersal in *P. aeruginosa* biofilms, NO was found to enhance cell motility, a phenomenon correlated with active dispersal. It was demonstrated that exogenous exposure to NO can induce dispersal in a broad range of biofilm-forming microorganisms and in complex communities of sessile microbes.^7,8^ One way of generating NO is to use the NO donor sodium nitroprusside. In aqueous solution, nitroprusside (SNP) readily decomposes to NO.^10,11^ The current work is motivated by biofilm detrimental effects and biocorrosion on metallic surfaces exposed to seawater found in marine heat exchangers and seawater handling systems.^1^ Existing inhibition strategies are costly and inappropriate, leading to microbe resistance and/or toxicity problems (i.e., toxic byproduct discharge in seawater). Developing an alternative means of controlling biofilms is a great challenge; however, there are benefits to be gained. The objectives of this work were to

1 use electrochemical impedance spectroscopy (EIS) at open-circuit potential (OCP) for sensing single-culture aerobic *Pseudoalteromonas sp*. biofilms on gold (Au) surfaces within a continuous culture flow cell and
2 explore the electrochemical performance of 72 h *Pseudoalteromonas sp*. biofilms on gold surfaces dosed with 500 nM NO donor SNP to induce NO-mediated biofilm dispersal.

## Experimental Section

### Flow Cell Arrangement

A once-through flow cell (5 × 6 × 40 mm) operated under a controlled low laminar flow condition (Reynolds number, *R*_e_ ≈ 1)^12,13^ using a Watson-Marlow series 323S peristaltic pump. The flow cell had a 0.2 mm diameter (area: 3.14 × 10^–4^ cm^2^) polycrystalline gold wire working electrode mounted on the upper surface to avoid gravitational effects, and a silver/silver chloride (Ag/AgCl) reference and graphite counter electrodes were mounted on opposing sides in the flow channel. The gold electrode was embedded in a glass cylindrical housing and had a surface roughness (*R*_a_) of 0.2 mm. The flow cell was under fully developed flow: an entrance length of 0.33 × 10^–3^ m for a 40 × 10^–3^ m channel length, a flow rate of 5.83 × 10^–9^ m^3^ s^–1^ (to minimize pulsative flow effects), a wall shear stress of 1.99 × 10^–3^ Pa, and a fluid residence time of 352 s^12,13^ The flow cell was designed to minimize abrupt fluid distortion that can lead to turbulence effects, see Supporting Information, Figure S1. A sealed 2 L reservoir pressurized with 0.2 μm filtered atmospheric air to supply the test media and a waste container were used. All components, tubing, and glassware were sterilized by either autoclaving or ethanol washes. After cleaning, these were rinsed thoroughly with 18.2 MΩ cm water. The flow cell was assembled within a laminar flow chamber, that is, under a particle-free and heated environment to perform microbiological or biotechnological procedures.

Supporting Information, Table S1, details the aerobic test media: (**1**) 3.5 wt.% NaCl; (**2**) artificial seawater (ASW) in accordance with Riegman et al. (dissolved salts and metal ions, vitamins, and nutrients),^14^ in addition, 0.1% (w/v) tryptone and 0.07% (w/v) yeast extract were added to enhance the ASW organic carbon content relevant to open seawater conditions;^15^ (**3**) ASW with a single aerobic *Pseudoalteromonas sp*. strain; and (**4**) ASW with 500 nM (0.13 ppm) of the NO donor SNP, where the freshly prepared SNP solution was kept at 0.12 ± 0.01 μmol photons m^–2^ s^–1^. The marine aerobic, bacterium *Pseudoalteromonas sp*. strain NCIMB 2021 was supplied by the National Collection of Industrial, Marine and Food Bacteria (NCIMB) in Aberdeen, UK. *Pseudoalteromonas* is a Gram-negative marine bacterium found in the open ocean and coastal seawaters, characterized as straight rods (length: between 2 and 3 μm; diameter: 0.5 μm) with a single polar flagellum, and utilizes carbon substrates: carbohydrates, alcohols, organic acids, and amino acids.^16^*Pseudoalteromonas* is a pioneer in terms of bacterial attachment and biofilm formation. The abiotic ASW was sterile and free of living organisms but contained carbon substrate/nutrients, whereas the biotic ASW was a more complex medium containing both nutrients and living organisms.^12,13^ The test solution using 500 nM SNP (NO donor) in ASW was also biotic, where *Pseudoalteromonas sp*. biofilms were allowed to grow on the gold electrode surface.^13^ All test media were freshly prepared using 18.2 MΩ cm water, 0.22 μm filtered, and had dissolved oxygen (DO) levels between 6.9 and 7.0 parts per million (ppm). A new cell culture was resuscitated from a freeze-dried ampoule and subcultured twice in 20 mL of solid agar NCIMB medium 210 using sterile Petri dishes at 18 ± 1 °C over 72 h. A continuously aerated and stirred sterile standard batch culture containing 250 mL of agar NCIMB medium 210 was used to prepare 200 μL aliquots of 2 h *Pseudoalteromonas* culture (inoculum) for the electrochemical experiments and represented a bacterial growth phase with a planktonic cell population of approximately 3.5 × 10^6^ cells mL^–1^. The pH 7.3 agar solution for the bacterial culture initially contained in 1 L: 3.0 g of yeast extract, 5.0 g of tryptone, 15.0 g agar, 0.750 L of 0.22 μm filtered aged seawater from the Southampton Water UK and kept 4 months in the dark in a temperature-controlled room at 6 ± 1 °C, and 250 mL of 18.2 MΩ cm water. The physicochemical properties of the test media in Supporting Information, Table S1: conductivity, DO, pH, and temperature were assessed before and after each test. The conductivity (approx. 50 mS cm^–1^ at 18 °C), DO level (≈6.90 ppm), and pH (8.0 ± 0.2) were measured using an ATI Orion model 162 conductivity meter, a Hanna Instruments HI9145 DO probe with the media flowing at 0.3 m s^–1^ and a Hanna Instruments HI98129 Combo probe, respectively. Experiments were performed at 18 ± 1 °C. Overall, the physicochemical properties were representative of surface sea waters from the North Atlantic.^17^

### Electrochemical Measurements

EIS for biofilm growth was performed over 72 h at 18 ± 1 °C using a Gamry Instruments Ref600 potentiostat and EIS300 software at the OCP. After NO doping, an additional 24 h allowed time for the biocide inhibition studies. The applied sinusoidal potential was 10 mV_rms_, with a frequency range of 0.1 Hz–100 kHz. All electrochemical tests were performed in a Faraday cage to minimize interference due to external electromagnetic fields and light irradiance measured at 0.12 ± 0.01 μmol photons m^–2^ s^–1^. In contrast to common physical models reported for polymer and/or protective coatings, no consensus has been reached for the equivalent circuit (EC) modeling of biofilmed metallic surfaces since the interfacial mechanisms are complex. The Supporting Information provides an overview of the EIS assessment method and the EC model for a thin microbial film or a conditioning film (i.e., an adsorbed organic layer) on the gold surface. Gamry EChem Analyst software was used to analyze the EIS measurements. All interfaces using the gold electrode in chloride media were modeled using mass-transfer and charge control kinetics.^18,19^ Standard procedures for the selection of EC best-fit were followed: (i) the chi-squared (χ^2^) error was suitably minimized (χ^2^ ≤ 10^–4^) and (*ii*) the errors associated with each element were ranged between 0 and 5%.^20^ Previous studies have shown the interfacial capacitance, that is, a derived EIS parameter, is informative of bacterial attachment on sensor surfaces;^18,19,21−23^ particularly changes in capacitance are related to sessile bacteria coverage.^21^ The hypothesis can provide data of the overall interfacial adsorption processes, broadly assuming

3 no deconvolution between the biofilm and conditioning film,^21,24^
4 four electrons for the predominant ORR reaction (*z* = 4), and
5 the total surface charge (≈10^–12^ C per adhered bacterium) is accepted for a sessile bacterium.^25,26^ The exact value is dependent on bacterial strain and ionic strength within the cell wall and whether the charge transfer occurs from or to the bacterial cell surface.^25,26^

### Confocal Microscopy Characterization

The gold electrodes were removed from the flow cell and washed in 0.22 μm filtered sterile test media to remove any nonadherent bacteria cells. A Leica TCS SP2 laser scanning confocal microscope, with a Live/Dead BacLight molecular probe^27^ (from Invitrogen Ltd, Paisley, UK) at an excitation wavelength of 470 nm, was used to assess the bacterial colonies, the distribution in the EPS matrix, and to corroborate the sensor performance.^13^

## Results and Discussion

### Estimation of the Number of Sessile Bacterial Cells

**Figure 1:**
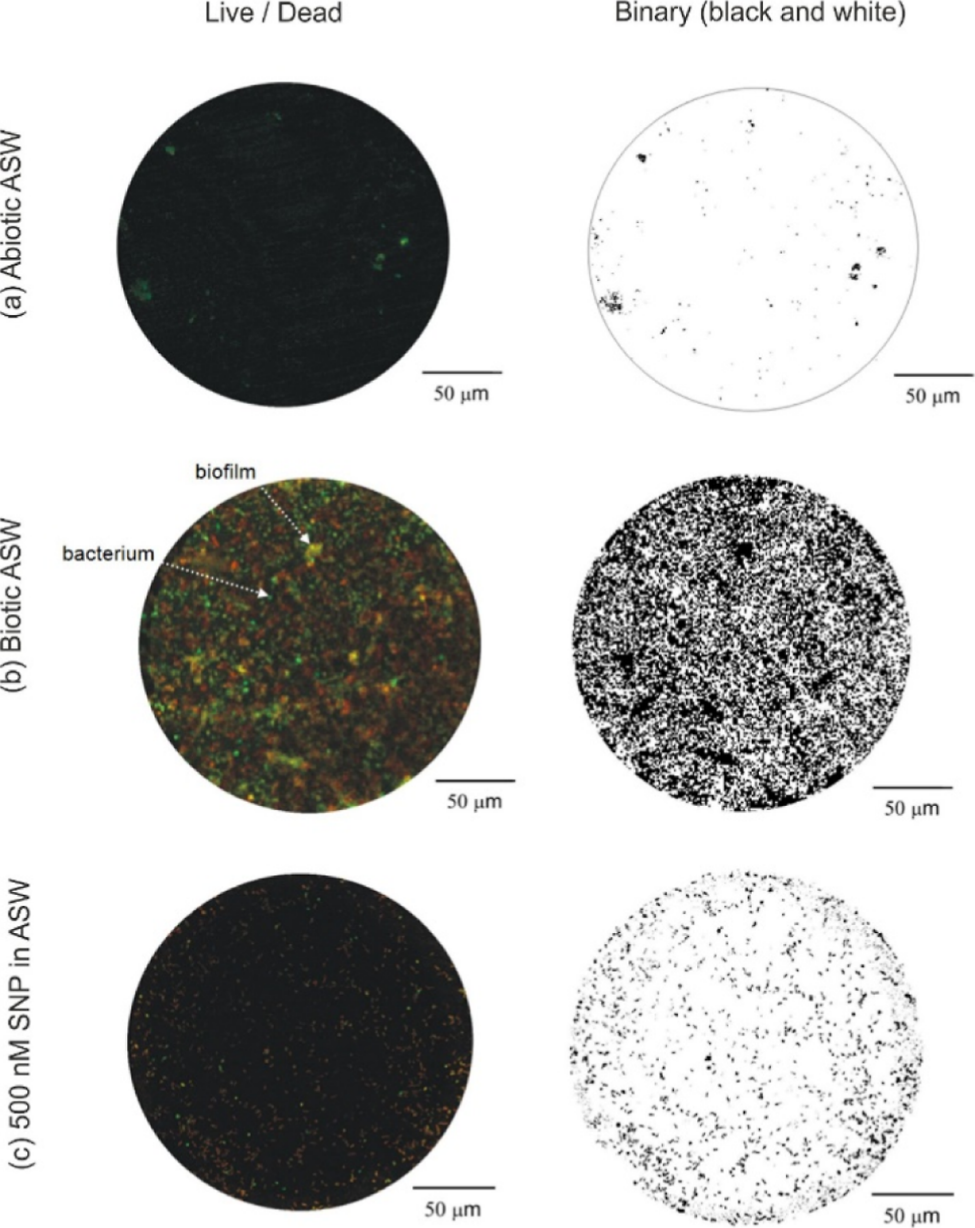
Confocal microscopy of a gold electrode stained with a BacLight viability kit and the corresponding binary (black and white) images utilizing ImageJ after 72 h immersion in **(a)** abiotic and **(b)** biotic ASW and also for **(c)** a 72 h biofilmed gold electrode after a 24 h immersion using 500 nM of the NO donor SNP.

Confocal microscopy of the gold electrochemical sensor after 72 h in sterile abiotic ASW (Figure 1a) displays indistinct colored spots often attributed to nonspecific binding (where stains bind to receptors that are nonbiotic in order to achieve a more favorable chemical configuration).^28^

In contrast, after 72 h in biotic ASW (Figure 1b), there is evidence of biofilm growth with bacterial microcolonies and a patchy distribution of EPSs (PI stains environmental DNA present in the EPS^29^), thus verifying bacterial colonization and biofilm formation.^1^ There is no evidence of flow orientation of the biofilm structures, contrasting with filamentous/streamlined biofilms often seen for turbulent flows.^20^ Pioneering bacteria will initially interact and adhere to the conditioning film. Colonization, growth, and EPS secretion will follow the initial bacterial adhesion using the available nutrients within the bulk test media. In the EPS, enzymes such as catalase can alter ORR kinetics within the biofilm.^13,30^ Importantly, the occurrence of bacterial clusters and EPS in Figure 1c was significantly reduced on the gold electrode surface after exogenous NO treatment. In this instance, the remnant biofilm consists of individual dead and/or damaged bacterial cells (cluster size and EPS have been markedly reduced). This confirms continuous exposure to low, nontoxic NO concentrations can induce effective and efficient dispersal in marine bacterial biofilms of *Pseudoalteromonas sp*..^7,8^ The binary images in Figure 1 were employed, utilizing the typical size of a single *Pseudoalteromonas sp*. cell, to estimate numbers of sessile bacteria and cell density on the gold surface after 72 h in the test media, see Table 1.

**Table 1:** Sessile Bacterial Cells for the Biotic ASW (72 h Biofilmed Gold Surface) and an Exogenous 24 h Exposure to 500 nM SNP in Biotic ASW (Area Covered by a Single *Pseudoalteromonas sp*. Cell of 0.95 ± 0.55 × 10^–8^ cm^2^: Using Cell Dimensions in Ref (31))

		cells/electrode surface	cells/10^6^ cells cm^–2^
sessile cells for complete surface coverage on the electrode surface		49,740 ± 28,800	160 ± 90
abiotic ASW (Figure 1a)^a^	live	225 ± 100	0.7 ± 0.3
	dead	350 ± 50	1.1 ± 0.2
	live/dead	250 ± 250	0.8 ± 0.8
biotic ASW	live	28,100 ± 1990	90 ± 6
(Figure 1b)	dead	24,175 ± 2485	77 ± 8
	live/dead	25,765 ± 1740	82 ± 5
500 nM SNP in ASW (Figure 1c)	live	3210 ± 150	10.2 ± 0.5
	dead	2510 ± 150	8.0 ± 0.5
	live/dead	3035 ± 250	9.6 ± 0.8

a Minimum fouling in an abiotic medium; however, a quantifiable nonspecific binding can be deduced for comparative purposes.

Confocal images for the four test media can be found in the Supporting Information Overall, confocal microscopy indicates the biofilms were a collection of both live and dead cells, which is consistent with the reported *Pseudoalteromonas* lifespan of roughly 72 h.^15^ As expected, greater cell numbers and densities were found on the gold surface exposed to the biotic ASW medium, where the biofilm exhibited normal unhindered development and microcolony formation. In contrast, numbers of bacterial cells were markedly lower for surfaces subject to exogenous exposure of NO. Overall, there was a 10-fold decrease of the biofilm after treatment with the 500 nM NO donor SNP. It should be noted that quantification of cell numbers assumes the bacterial distribution/microcolonies within the biofilm form a single layer and not structured three-dimensional microbial community clusters. Confocal microscopy confirmed that the most extensive biofilm surfaces after 72 h in biotic ASW had a thickness of 3.0 ± 0.5 μm (correlating with the longest *Pseudoalteromonas* cell dimension). The biofilm thickness is nonuniform, which is associated with multiple factors such as cell orientation, cell density, and cell-to-cell distance or distance between microcolonies. Within these limitations in assessing the exact numbers of attached bacterial cells, confocal microscopy provides relevant quantifiable data to further support the EIS results.

### Electrochemical Impedance Spectroscopy

**Figure 2:**
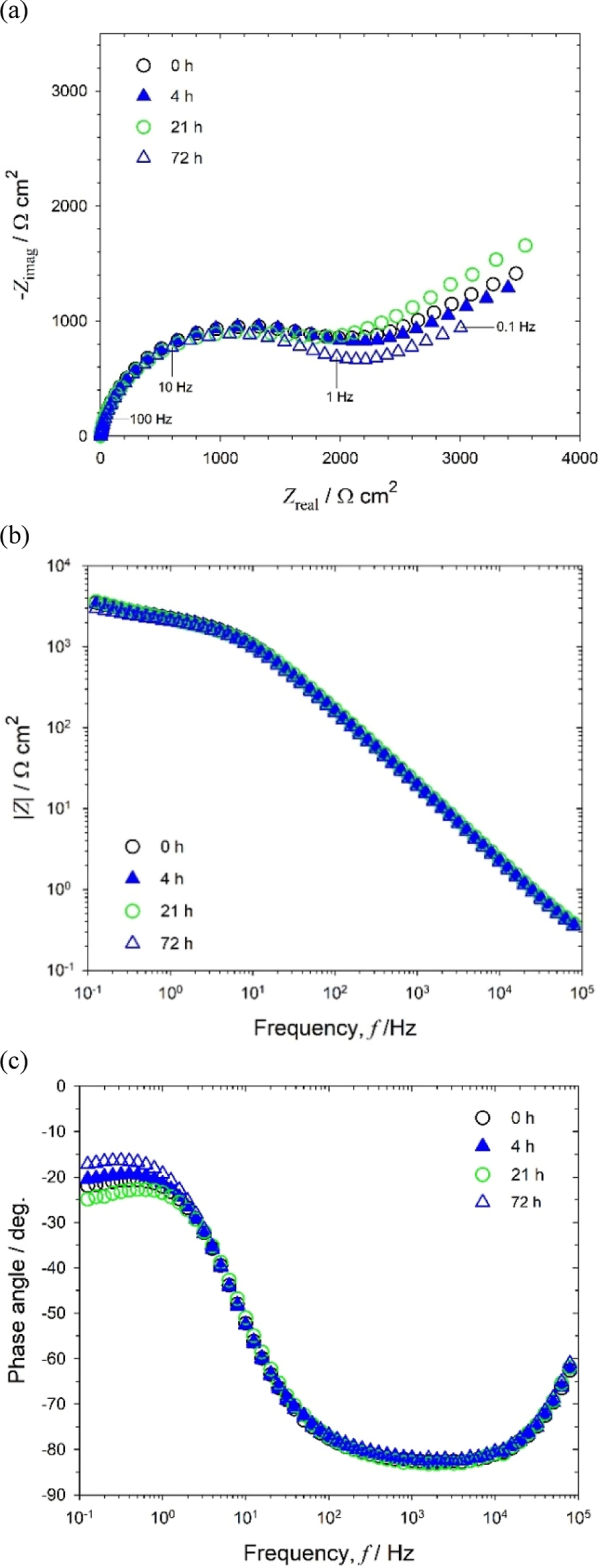
EIS for abiotic 3.5 wt % NaCl medium (#1): **(a)** Nyquist, **(b)** Bode |*Z*|, and **(c)** Bode phase angle for a 0.2 mm diameter gold electrode at OCP: +0.090, −0.070, −0.215, and −0.225 V after 0, 4, 21, and 72 h of immersion, respectively.

**Figure 3:**
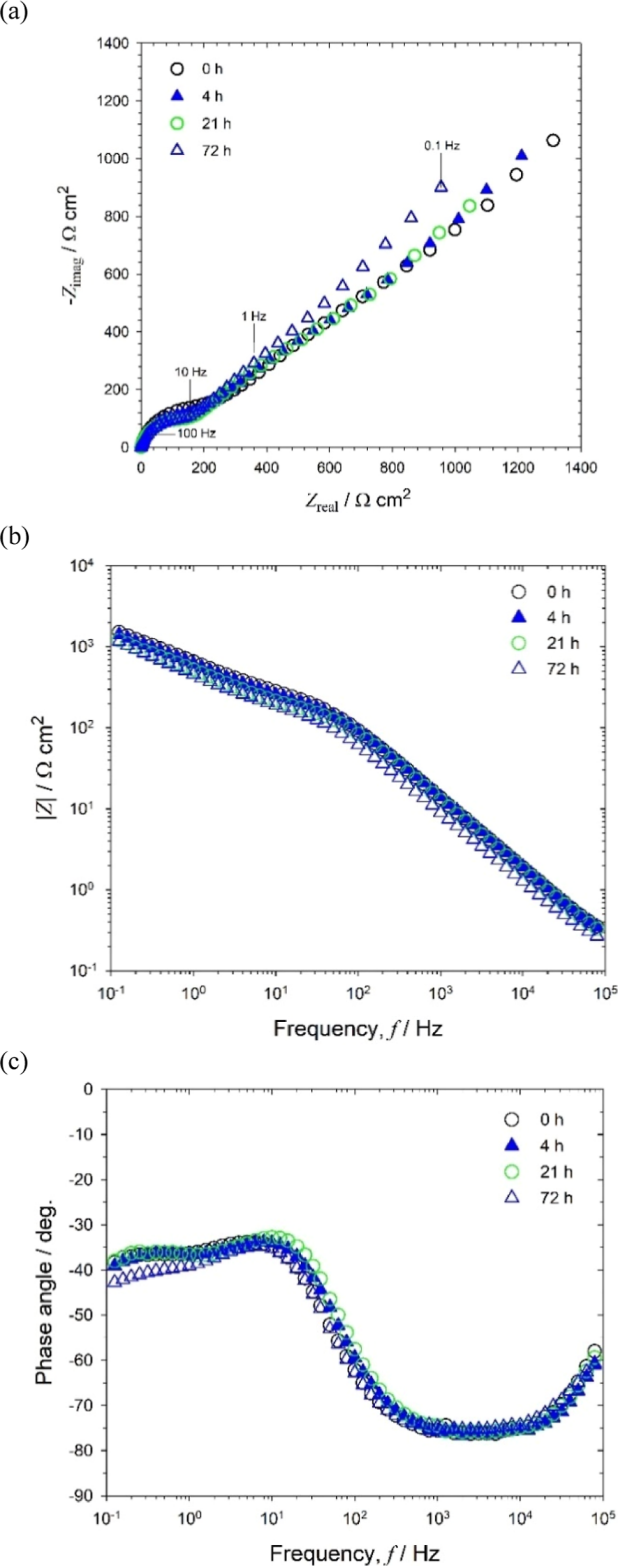
EIS for abiotic artificial seawater medium (#2): **(a)** Nyquist, **(b)** Bode |*Z*|, and **(c)** Bode phase angle at OCP: +0.085, −0.070, −0.250, and −0.325 V after 0, 4, 21, and 72 h of immersion, respectively.

**Figure 4:**
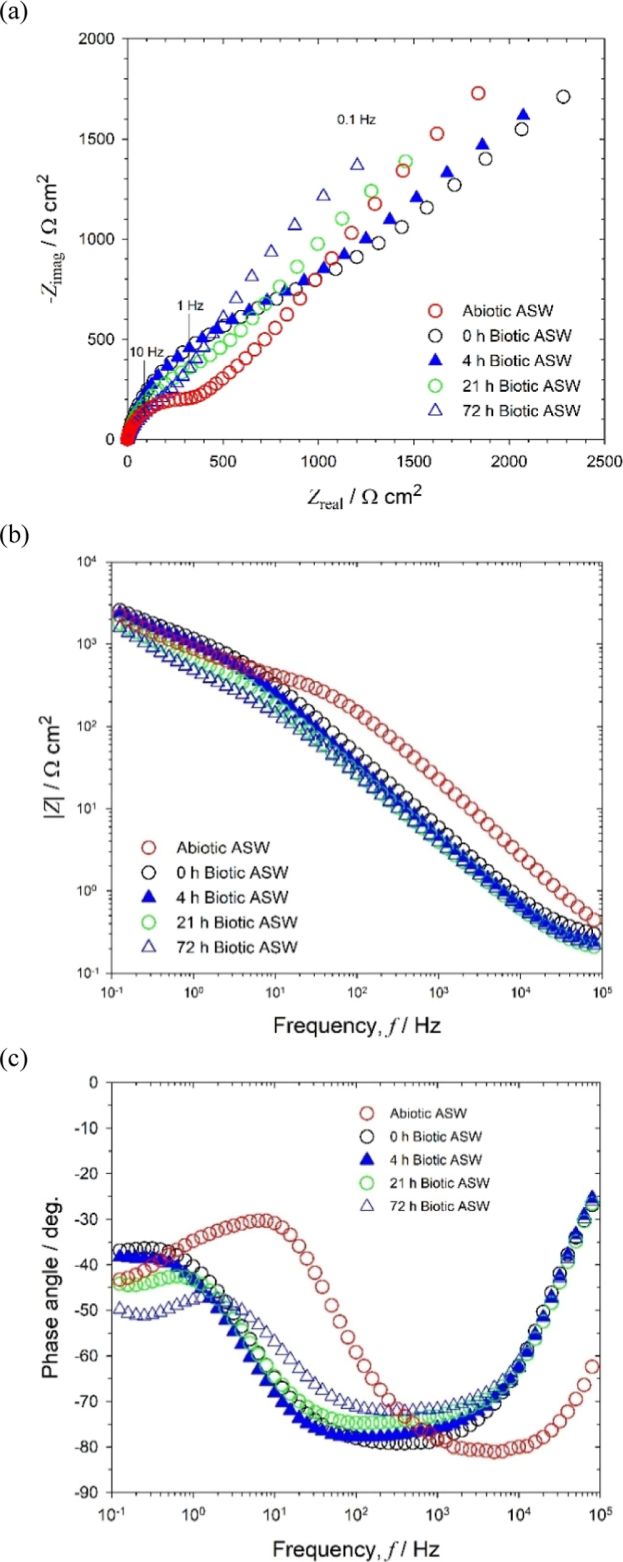
EIS for biotic artificial seawater medium (#3): **(a)** Nyquist, **(b)** Bode |*Z*|, and **(c)** Bode phase angle for a 0.2 mm diameter gold electrode at OCP: +0.090, +0.085, −0.075, −0.460, and −0.560 V at −1 h (abiotic) and after 0, 4, 21, and 72 h of immersion, respectively.

**Figure 5:**
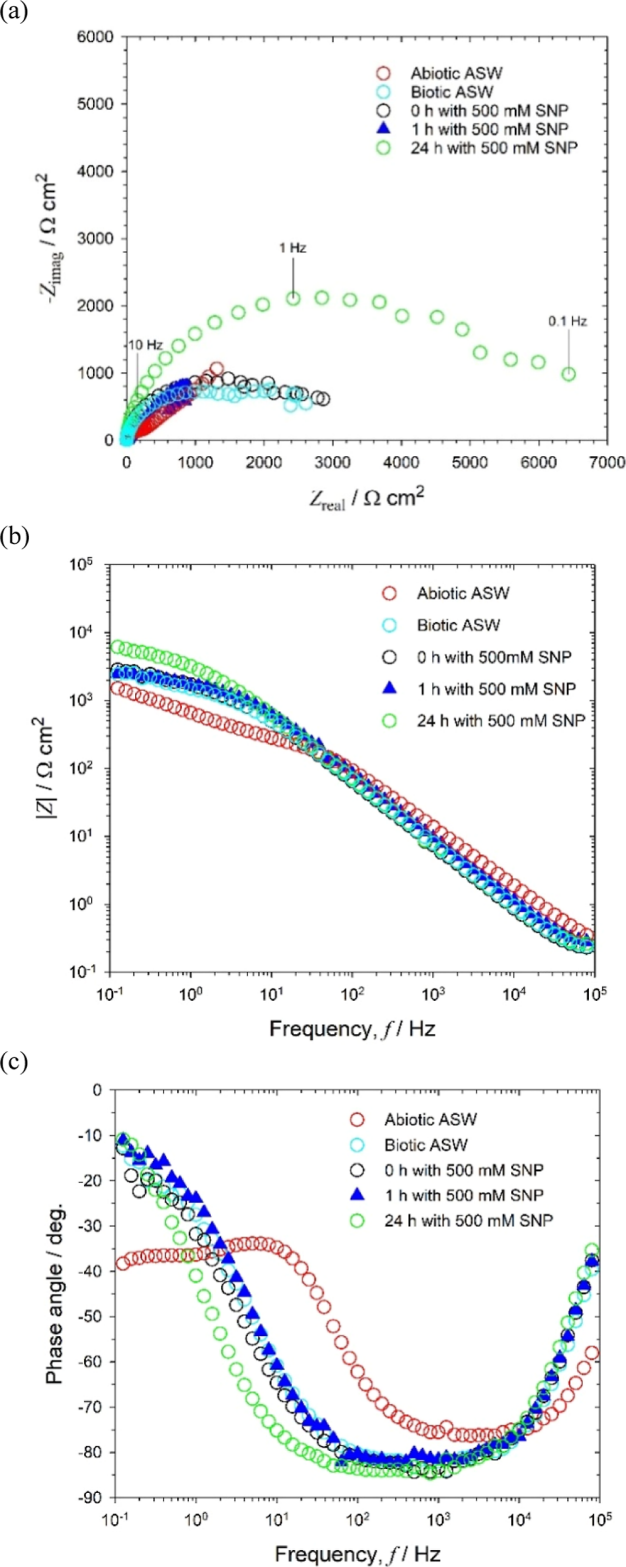
EIS for biotic artificial seawater (72 h) and exogenous NO exposure–medium #4: **(a)** Nyquist, **(b)** Bode |*Z*|, and **(c)** Bode phase angle for a 0.2 mm diameter gold electrode at OCP: +0.080, −0.470, −0.470, −0.470, and −0.325 V at −1 h (abiotic) and 72 h (biotic) after 0, 1, and 24 h with 500 nM SNP, respectively.

EIS results are presented in three forms (Figure 2 to 5): the Nyquist (imaginary vs. real components), Bode |*Z*| (|impedance| vs. frequency), and Bode phase angle (θ vs. frequency) plots. The impedance data for abiotic NaCl typifies the electrochemical performance of the gold electrode in a near-neutral/alkaline solution^32^ and allows comparison between the abiotic ASW and biotic ASW media to evaluate the sensor performance.

In addition, −*Z*_imag_ versus *f* plots were used to justify the constant phase element (CPE) parameters in the EC modeling (Supporting Information, Figure S2). This gives a better description of the capacitive behavior (used to determine *C*_eff_) since the slope can be associated with the CPE behavior.^33^ Overall, graphically derived parameters are presented alongside other EIS data in Supporting Information, Table S2. Figure 2 shows that the EIS for abiotic 3.5 wt % NaCl remained uniform with time with two distinct regions.

The high-frequency region, 10 Hz–100 kHz (Figure 2b), gives a linear relationship between the interfacial impedance modulus and frequency with a −1 slope, linked with a phase angle plateau close to −90° (Figure 2c) and partially resolved semicircles in Figure 2a. This is predominant capacitance behavior, consistent with studies using a similar sterile configuration^32^ that relates to the well-established double-layer concept (i.e., interfacial charge distribution).

Within this high-frequency region, the plots show good reproducibility. At lower frequencies, between 0.1 and 10 Hz, an additional resistance/diffusive response (Figure 2b) can be seen with minor variations in impedance over 72 h, thus demonstrating the absence of detectable changes with time. The diffusive behavior is associated with linear features having a 45° slope (a Warburg impedance response) in Figure 2a. This impedance behavior results from the diffusion of electroactive DO to the gold surface to participate in the ORR.^32^

Supporting Information, Figure S5 shows the confocal analysis of a gold electrode after 72 h of NaCl immersion, with the gold electrode surface nearly free of fouling and nonspecific binding. Overall, the EIS curve in the sterile abiotic ASW (Figure 3) tends to shift with time toward lower frequencies. This behavior is associated with interfacial adsorption dominant processes.^24^ In the high-frequency region, between 100 Hz and 100 kHz (Figure 3b), a slope close to −1 is evident corresponding to a plateau of the phase angle relatively close to −90° and to partially resolved Nyquist semicircles. This is a capacitance characteristic associated with a conditioning film due to rapid formation of an adsorbed layer of organic material on the gold surface.^2^ Typical conditioning film thicknesses are between 6 and 10 nm.^1^ In the low-frequency region, 0.1–100 Hz, a diffusive response can be seen in Figure 3b. This coincides with the presence of a delineated linear feature having a 45° slope in Figure 3a, which is characteristic of diffusion of dissolved species, such as electroactive DO. It should be noted that the organic components in the sterile ASW (medium #2) are B vitamins that are known to act as redox mediators.^34^ The enhanced reduction kinetics linked to the presence of an organic conditioning film, in which organic molecules (B vitamins and ethylenediaminetetraacetic acid) form a loosely packed monolayer on the gold surface, where discontinuities allow oxygen mass-transfer to continue.^35^ Again, the presence of indistinct and faint colored spots in Supporting Information, Figure S6 is representative of minimum fouling and explained by nonspecific binding.^28^

In comparison with sterile abiotic ASW, the EIS for the biotic ASW gives a more complex impedance response (Figure 4). The EIS for the initial abiotic ASW (−1 h, i.e., the immersion period prior to inoculation) is similar for both test media; however, after inoculation (0 h), the capacitive behavior extended deeper into the low-frequency region (Figure 4b,c) with an overall increase in the diameter of the resolved Nyquist semicircles in Figure 4a. This is indicative of a greater influence of adsorption processes,^24^ associated with the adhesion of the pioneering bacteria on a conditioning film,^18,22^ thus consistent with reference (36). At lower frequencies, 0.1–to 100 Hz (Figure 4b), a diffusive behavior with a subtle change in impedance, was observed compared to the abiotic condition. This coincides with a decrease of the depressed Nyquist semicircles with a tail having a slope close to 45°, see Figure 4a. This can be explained by ORR enhancement by enzymatic processes, thus correlating with similar work using the same strain on 70/30 cupronickel alloys (here, the corrosion products and oxide film formation also influenced the EIS response).^31^ Supporting Information, Figure S7 shows the confocal microscopy of a gold electrode after 72 h of immersion in biotic ASW. The bacterial microcolonies and patchy EPS are clearly seen, thus corroborating the presence of bacterial biofilms.^1^ The biofilm thickness after 72 h was 3.0 ± 0.5 μm, consistent with the formation of a thin physical diffusion barrier and similar to reported biofilms between 4 and 8 μm thickness on a gold surface after 10 days of exposure to seawater.^37^Figure 5 shows that the EIS measurements for abiotic and biotic conditions are qualitatively comparable with the electrochemical performances in Figure 4. These include the impedance shift toward lower frequencies (Figure 5b,c), the diffusive/resistive behavior, and the minor change in impedance (Figure 5b) in the low-frequency part of the spectra, 0.1–100 Hz. Initially at 0 h, immediately after NO dosing of a mature 72 h old biofilm, no marked change in impedance, was immediately apparent. Importantly, a detectable modification with an increase in the interfacial resistance at lower frequencies was evident after prolonged exogenous NO exposure. This can be explained by a significant suppression of the interfacial charge transfer resulting from biological stress induced by NO on the bacterial biofilms.^7,8^ Although the exact dispersal mechanism of NO action remains to be fully elucidated,^7,38^ the detectable increase in impedance can be linked with biofilm sloughing and dispersal. Likewise, subtle interfacial charge transfer (smaller depressed Nyquist semicircle after 1 h NO exposure to that for 0 h) in Figure 5a can account for the residual ORR via enzymatic processes. It is possible that adsorbed NO and DO together compete on electroactive sites, thereby affecting the gold interface. The confocal microscopy (Supporting Information, Figure S8) uniquely assesses the performance of exogenous NO exposure on 72 h *Pseudoalteromonas* biofilmed gold surfaces, revealing that bacterial microcolonies were greatly reduced on the gold electrode. These results are consistent with where significant antibiofilm effects (a decrease in biofilm biomass and an increase in planktonic biomass) were observed with 500 nM SNP.^39^

**Figure 6:**
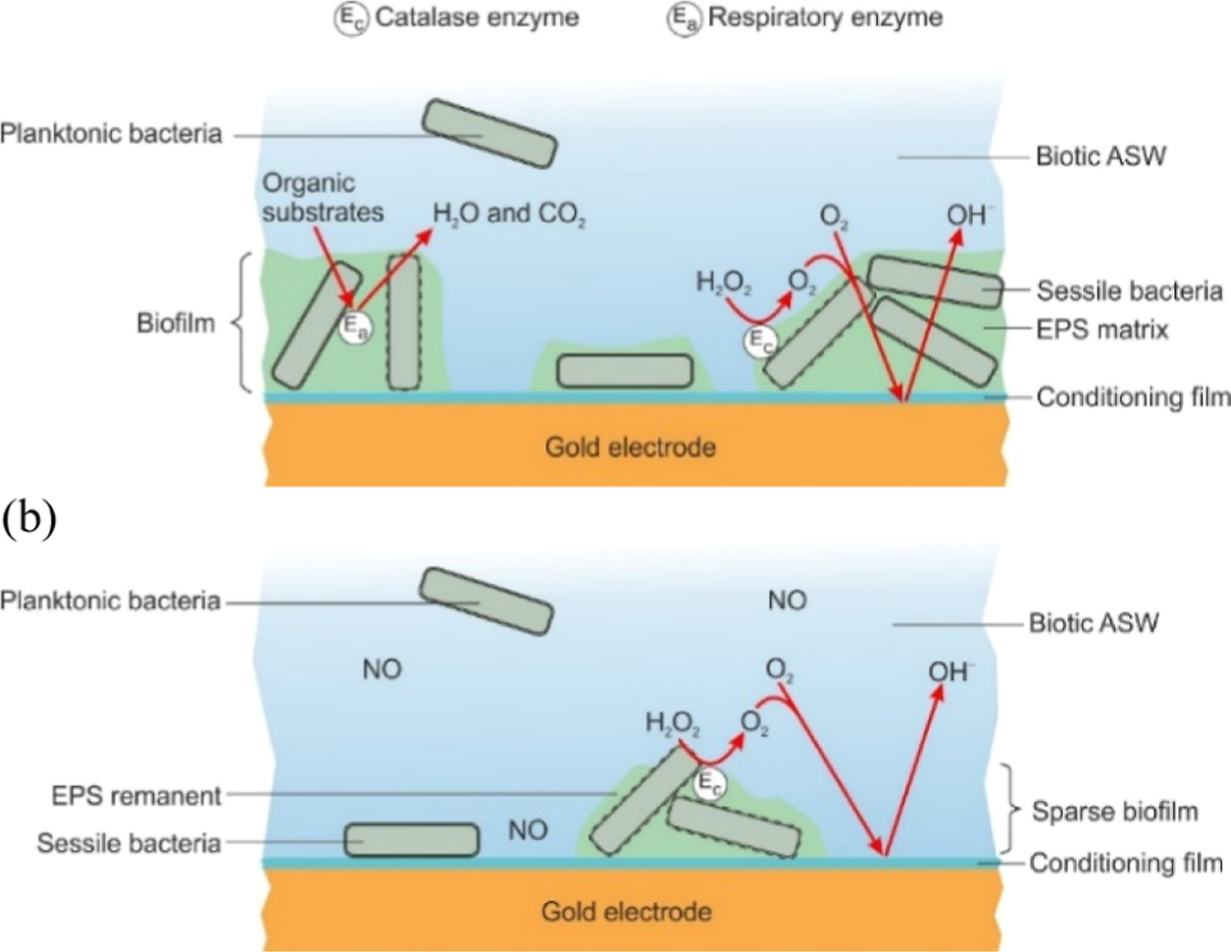
Schematic of electron-transfer pathways within a *Pseudoalteromonas* biofilm—typical thickness of the bacterial biofilm is between 2 and 3 mm: **(a)** biofilm growth and colonization under aerobic biotic ASW conditions and **(b)** biofilm dispersion/disruption for aerobic biotic ASW with a 500 nM NO donor.

Supporting Information, Table S2, shows that the initial 0 h EIS data (*R*_ct_ and *Y*_o_) for the abiotic and biotic ASW (#2, #3, and #4) are similar; however, after dosing with NO, the *R*_ct_ increased with a simultaneous decrease in capacitance where values reached 27.2 μF cm^–2^ (#4). Although the exact significance of the *C*_eff_ components remains to be fully elucidated,^40^ the capacitance decrease can be explained by a degradation of the biofilm exposed to NO (marked decrease in biofilm biomass), where affected and lysed bacteria modify the biofilm.^18^ Likewise, the *R*_ct_ increase after 24 h in the presence of NO (which is higher than that for abiotic NaCl) is indicative of suppressed interfacial charge transfer due to a physical modification of the interface, for example, sparse biofilm remnants and individual dead bacteria confirmed by the confocal analyses.^12,13^ Finally, from the surface charge density, an estimation of the number sessile bacteria can be done. Figure 6 illustrates the electron-transfer pathways during biofilm colonization and for biofilm dispersal (dead sessile bacterial cells physically block the electrode redox processes). When bacterial biofilms develop on the initial conditioning film, a DO concentration gradient is generated (differential aeration cell), leading to mixed mass-transfer and charge control kinetics.^31^ Consequently, bacteria assist in electron transfer, where the most active cells can be found at the biofilm/seawater interface.^41^ Overall, the enzymatically enhanced ORR via catalase enzymes is the prevailing reaction at cathode sites (Figure 6), which will be dependent on the EPS extent. Lower-catalase H_2_O_2_ scavenging leads to higher H_2_O_2_ concentrations as the ORR on gold proceeds via an intermediary mechanism.^42−45^ Barraud et al. reported that exposure to 500 nM SNP significantly enhanced the efficacy of antimicrobial compounds, for instance hydrogen peroxide, in the removal of established biofilms.^7^ Accordingly, the combined exposure to both NO and the intermediary ORR antimicrobial agent (H_2_O_2_) may therefore offer a novel strategy to control persistent marine biofilms. The Warburg diffusion term (*W*) is not reported, although *W* is presented in Supporting Information, Tables S3–S6. Likewise, the *R*_s_ for the various test media ranged between 0.163 and 0.170 Ω cm^2^, thus corresponding to conductivities where σ = 47.0 ± 1.0 mS cm^–1^ at 18 °C for 35‰ salinity and demonstrate the nominal effect of the external reference capacitance (i.e., a minimum variation of the *R*_s_ component explained by a negligible influence of the *C*_ref_ parameter on the measurements in Supporting Information, Table S2.

### Bacterial Biofilm Sensor Relationship

**Figure 7:**
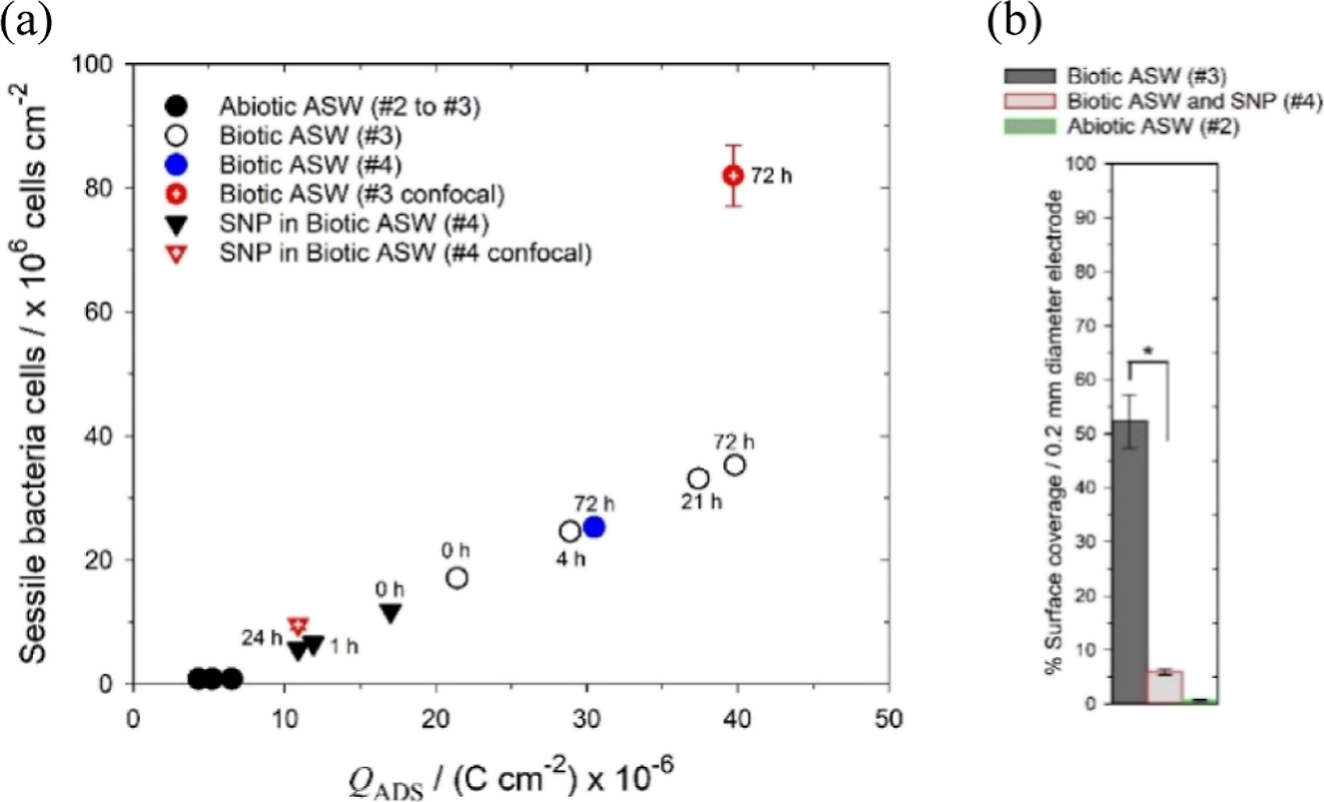
**(a)** Number of sessile *Pseudoalteromonas sp*. cells vs surface charge density for test media (#2 to #4) – red symbols with a crosshair are the ex situ confocal microscopy assessment of the number of live and dead bacterial cells (Table 1). **(b)** Bacterial surface coverage analysis for abiotic, biotic ASW, and biotic with 500 nM SNP (*p < 0.05 via Student’s *t*-test).

After inoculation (0 h), Figure 7a shows the EIS-determined surface charge density for the biotic ASW increases (i.e., due to formation of an adsorbed organic layer and pioneering bacterial adhesion on the gold) ranges between 21.4 and 39.7 μC cm^–2^ over 72 h of immersion, thus associated with the bacterial biofilm growth. Assessment of the number of sessile bacterial cells for the 72 h biofilmed gold surface (Supporting Information, Table S2) is 35 × 10^6^ cells cm^–2^, which is equivalent magnitudinally with the 82 × 10^6^ cells cm^–2^ determined from ex situ confocal microscopy in Table 1 and Figure 1b. Although similar in magnitude, the difference observed between the (medium #3) 72 h biotic ASW data (39.7 μC cm^–2^) and (media #4) (30.4 μC cm^–2^) in Figure 7a was expected due to stochastic/random effects incurred by biological systems and the presence of different gold surface active areas per testing. In contrast, the charge density decreased upon NO exposure associated with bacterial biofilm dispersal. After 24 h, the EIS-determined number of sessile bacterial cells for the NO in ASW was 5.7 × 10^6^ cells cm^–2^ (Figure 7a), which is in good agreement with the 9.6 × 10^6^ cells cm^–2^ via confocal microscopy (Table 1 and Figure 1c). A comparative study (percentage surface coverage) of the biofilm extent on gold electrode surfaces in both abiotic and biotic ASW, as well as biotic ASW with SNP, is shown in Figure 7b. Whereas the biofilm for abiotic ASW is negligible (close to a few percentage—linked to nonspecific binding), a significant biofilm coverage has accumulated on the gold surface in biotic ASW. Overall, confocal microscopy (Figure 1) for the biotic conditions shows that the biofilm is composed of live and dead cells. After NO exposure, a marked decrease in the biofilm from about 50 to 5% was observed. This indicates that the surface charge density can be utilized to quantitatively inform the presence of bacterial biofilms, where the capacitance can be the main component of the measurement procedure.

**Figure 8:**
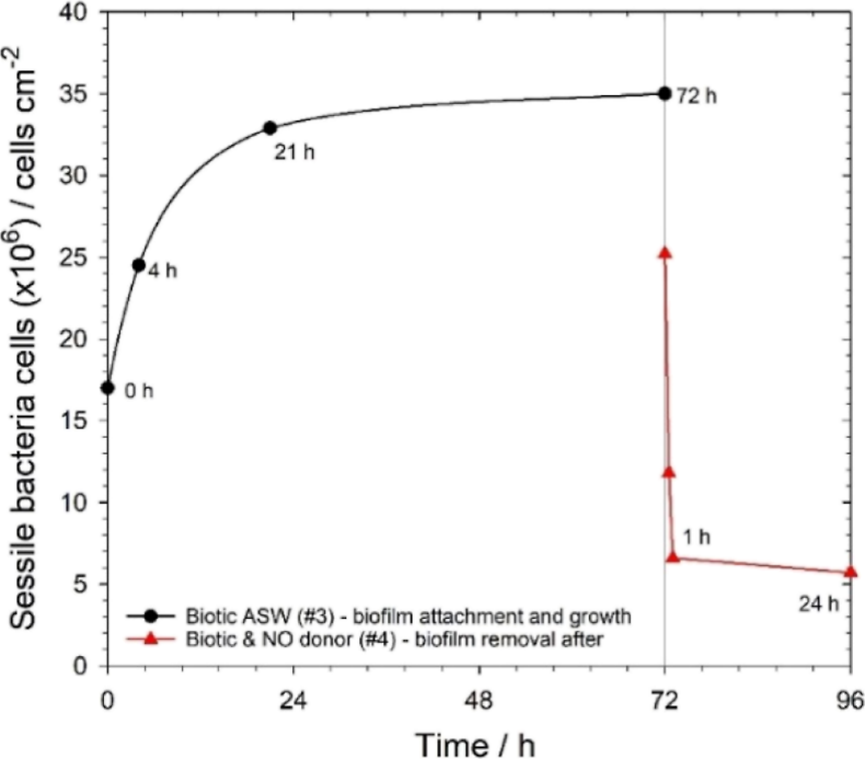
EIS-derived sessile bacterial density vs time under low laminar flow. Biotic ASW over 72 h and after 24 h prolonged exposure to 500 nM SNP (NO donor) in the biotic ASW medium.

Figure 8 shows that the EIS-derived sessile cell density rapidly increases for the biotic ASW test medium associated with biofilm attachment and colonization within the first 24 h, followed by steady-state growth and dispersal for the next 48 h. Once the mature 72 h biofilm is exposed to low NO concentrations, there is a marked decrease in cell density within the first hour and remained at these low levels for the following 24 h. The EIS-derived assessment of bacterial numbers demonstrated differences between abiotic and biotic media. The qualitative EIS analyses have shown initial marine bacterial biofilms can be electrochemically detected under a controlled flow cell environment. Importantly, a key EIS parameter is the capacitive component (between 100 Hz and 100 kHz), which can be used to quantify biofilm and subsequently gauge the biofilm extent. Uniquely, the number of attached bacterial cells on the gold surface (after 72 h of biofilm growth and 24 h of exposure to NO estimated from the impedance data, in situ sensing) was in relatively good agreement with an ex situ assessment using confocal microscopy analyses. This supports the relationship between the surface charge density induced by biofilm and the corresponding sessile bacterial population, providing insights into sensor calibration used in real-time biofilm-monitoring devices.

Similarly, the EIS response after NO dosing the biotic ASW uniquely demonstrated a significant impedance change, which was corroborated using confocal microscopy. This was indicative of an effective and efficient biofilm dispersal using low, nontoxic concentration of the NO donor, therefore consistent with refs (7) and (8). These considerations support the studies on the antifouling efficiency and the molecular mechanisms inherent to the nanomolar-range NO donor SNP dosed on a metal surface. Little is known about the main factors that can influence the bacterial dispersal mechanisms due to chemical stresses.^38^ In addition, it was shown that the biocide concentration is a critical parameter for biofouling control.^7,46^ For instance, concentrations below the threshold required to inhibit growth can enhance biofilm formation.^46^ In this study and as proposed by Barraud et al.,^7^ 500 nM SNP can be the optimum concentration to clean a substratum; that is, a metallic surface. In practice, the duration (ideally short) and frequency of dosing (continuous dosing, shock treatment, and pulse dosing) and also the flow regime are of great importance to define suitable dosing strategies for biofilm control.^46^ Ideally, an intelligent combination of these three factors should be addressed to maintain the operating systems and reduce the capital costs incurred. However, it is still unclear how this can be achieved since biofilms are competent biological systems that can constantly adapt to extremely different environmental conditions.^1,2^

## Conclusions

This study quantified sessile *Pseudoalteromonas* cells and biofilm coverage on a 0.2 mm diameter gold electrode utilizing an EIS-derived surface charge density parameter. Surface charge density allows the evaluation of adhered cell numbers corroborating with ex situ confocal microscopy. Key insights include the following:

1 EIS for abiotic NaCl was uniform with time, demonstrating a capacitance response at higher frequencies (interfacial charge distribution) and a diffusive/resistive characteristic at lower frequencies (linked to DO diffusion);
2 sterile abiotic ASW gave a capacitance response at higher frequencies (adsorbed organic layer and/or conditioning film) and a diffusive response at lower frequencies;
3 EIS for biotic ASW was more complex with an extension of the capacitive region at higher frequencies (greater adsorption processes/adhesion of pioneering bacteria) and a diffusive behavior and change in impedance over 72 h at lower frequencies (enzymatically enhanced ORR); and
4 the qualitative EIS/confocal microscopy-confirmed bacterial biofilm growth and extent are a dynamic and complex process, where pioneering bacteria will initially adhere and colonize on the gold surface to subsequently secrete EPS and favor enzymatic reactions for the enhanced ORR.

Overall, this study demonstrates that components of the EIS response (i.e., the capacitance parameter between 100 Hz and 100 kHz) provide quantifiable data that inform the extent of biofilm adhesion and colonization on the gold electrode. Different biofilm species and strains will likely have different values due to biophysical differences in their cell structure and physiology.

## Supporting Information Available

Additional experimental details and methods, including confocal microscopy and graphical/tabulated EIS data (PDF)
