# Supplementary material for: Monitoring Aerobic Marine Bacterial Biofilms on Gold Electrode Surfaces and the Influence of Nitric Oxide Attachment Control
Source: Anal Chem. 2022 Aug 31;94(36):12323–32. doi: 10.1021/acs.analchem.2c00934 (PMC9475501; doi:10.1021/acs.analchem.2c00934)
Supplement: Supplementary file 1 — ac2c00934_si_001.pdf [file ac2c00934_si_001.pdf]

Supporting information for

Monitoring aerobic marine bacterial biofilms on gold electrode surfaces and the influence of nitric oxide attachment control

Stephane Werwinski[a], Julian A. Wharton[a]*, Mengyan Nie[a,b], Keith R. Stokes[a,c]

[a] National Centre for Advanced Tribology at Southampton (nCATS), Faculty of Physical Sciences and Engineering, University of Southampton, Highfield, Southampton, SO17 1BJ, UK.

[b] UCL Institute for Materials Discovery, University College London, Malet Place, London, WC1E 7JE, UK.

[c] Physical Sciences Department, Dstl, Porton Down, Salisbury, Wiltshire, SP4 0JQ, UK.

* Corresponding author: j.a.wharton@soton.ac.uk

NOMENCLATURE

TERM	MEANING	UNIT
C_{eff}	Effective capacitance	F cm^{-2}
C_{ref}	External reference capacitance	F cm^{-2}
f	Frequency	Hz
F	Faraday constant	96,485 C mol^{-1}
j_{OCP}	Current density at the-open circuit potential	A cm^{-2}
i	Imaginary number	dimensionless
n	Empirical exponent of the constant phase element (CPE)	dimensionless
P	P-value	dimensionless
Q_{ads}	Surface charge density	C cm^{-2}
$Q_{ads(abiotic)}$	Surface charge density for the abiotic condition	C cm^{-2}
$Q_{ads(biotic)}$	Surface charge density for the biotic condition	C cm^{-2}
r^2	Square of the sample correlation coefficient	dimensionless
R	Molar gas constant	8.314 J K^{-1} mol^{-1}
R_{ct}	Charge transfer resistance	Ω cm^2
Re	Reynolds number	dimensionless
R_s	Solution resistance	Ω cm^2
T	Temperature of the solution	K
ω	Angular frequency	rad s^{-1}
Y_o	Admittance corresponding to (1/|Z|) at ω = 1 rad s^{-1}	Ω^{-1} cm^{-2} s^n
z	Number of electrons involved in an electrochemical reaction	dimensionless
Z_{CPE}	Constant phase element impedance	Ω cm^2
Z_W	Warburg impedance	Ω cm^2
σ	Electrical conductivity	mS cm^{-1}

SUPPORTING INFORMATION

Additional experimental and analysis methods are provided here, including flow channel and electrochemical details, confocal microscopy and graphical/tabulated EIS data.

(a)

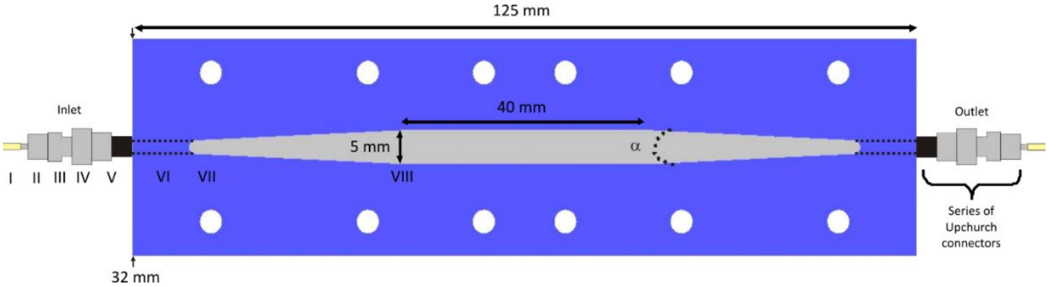

(b)

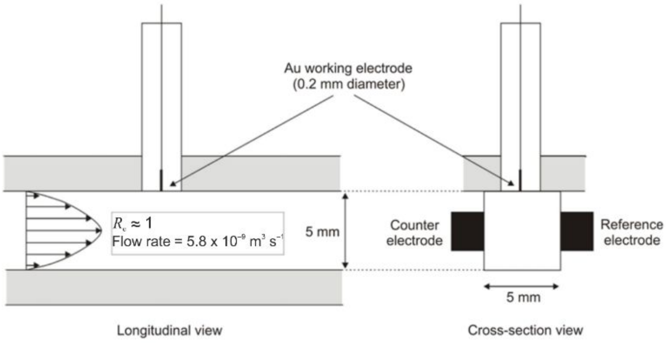

Figure S1. **(a)** Plan view of the flow channel showing the included angle, α = 6° and connectors; **(b)** Schematic of the flow cell electrode arrangement for the electrochemical measurements. Connectors: **I** – Marprene tubing (Ø 0.63 mm bore), **II** – P-692 (Ø 0.5 mm through-hole), **III** – P652 (Ø 0.75 mm through-hole), **IV** – P669-01 (Ø 1.25 mm through-hole), **V** – P-672+10-32 FE NanoPort (Ø 1.25 mm through-hole), **VI** – Ø 2.00 mm through-hole, **VII** 3.0 mm and **VIII** 5 mm.

Electrochemical assessment. In analogy with electrochemical investigations of protein adsorption on metallic surfaces,[1-5] the relationship between the effective capacitance (C_{eff}) and surface charge density (Q_{ADS}), can be expressed by Eqn. S1 assuming a minimum variation of the local resistivity can be given by:[4, 6, 7]

$$Q_{\mathrm{ADS}} = \frac{4zRT}{F} C_{\mathrm{eff}} \quad \text{......(S1)}$$

where z is the number of electrons involved in the electrochemical reaction, R = 8.3144 J K^{-1} mol^{-1} the molar gas constant, T the temperature of the test solution in K and F = 96485 C mol^{-1} the Faraday constant. Effective capacitance is defined using Eqn. S2[6, 7], where R_{f} represents the film resistance, n is the empirical exponent of the constant phase element (CPE) and Y_{o} has the numerical value of the admittance ($1/|Z|$, with units of Ω^{-1} cm^{-2} s^{n}) at ω = 1 rad s^{-1}.

$$C_{\mathrm{eff}} = (R_{\mathrm{f}})^{(1-n)/n} Y_{\mathrm{o}}^{1/n} \quad \text{......(S2)}$$

The C_{eff} parameter in reality does not behave ideally as a capacitor due to non-homogeneity behaviors linked to surface roughness,[8] distribution of reaction rates[9], varying thickness or composition[10] and/or non-uniform current distribution[11, 12]) and consequently a CPE[13, 14] is used to model these components. The CPE impedance may be defined by:

$$Z_{\mathrm{CPE}} = \frac{1}{Y_{\mathrm{o}}(i\omega)^{n}} \quad \text{......(S3)}$$

The exponent parameter n usually lies between 0.5 and 1. When n = 1, the CPE describes an ideal capacitance, where in this instance Y_{o} is representative of the capacitance. For 0.5 < n < 1, the CPE represents a distribution of dielectric relaxation times in frequency space. When n = 0.5, the CPE defines a Warburg impedance which provides information on diffusion behavior [13, 14]. The similarity between the impedance derived capacitor and CPE parameters makes it tempting to approximate Y_{o} as an ideal capacitance when n approaches unity; however, this is an inappropriate approximation since Y_{o} does not have units of capacitance and relatively small deviations of n from unity can lead to significant errors.

The film resistance in Eqn. S2 can correspond to the charge transfer resistance (R_{ct}) in Eqn. S4 for a thin bacterial biofilm, where the R_{ct} parameter indirectly relates to the baseline ORR ($O_2 + 2H_2O + 4e^- \longrightarrow 4OH^-$) in alkaline media and/or the enzymatic enhanced cathodic reduction involving electron transfer at the gold/biofilm interface, or redox-active center within the EPS matrix/seawater interface.[13, 15] The ORR on gold proceeds only via an intermediary mechanism, with H_2O_2 as the reactant intermediate. In alkaline solutions, oxygen is readily reduced to H_2O_2 and only partial peroxide reduction to water occurs on the gold surface ($O_2 + 2H_2O + 2e^- \rightarrow H_2O_2 + 2OH^-$).[16-19]

$$R_{\mathrm{ct}} = \frac{RT}{zFj_{\mathrm{OCP}}} \quad \text{......(S4)}$$

where j_{OCP} is the current density at the open-circuit potential (OCP). Using the reported total surface charge of various bacterial strains defined by proton titrations ($\approx 10^{-12}$ C per adhered bacterium[20, 21]) involved in current exchange during adhesion, Eqn. S1 can be exploited to address a relationship of the surface charge density of sessile cells to their adhered population (after subtraction of the abiotic response) using Eqn. S5:

$$\text{Number of cells} \cong \frac{Q_{\mathrm{ADS}_{\mathrm{(biotic)}}} - Q_{\mathrm{ADS}_{\mathrm{(abiotic)}}}}{10^{-12}} \quad \text{......(S5)}$$

where $Q_{\mathrm{ADS}_{\mathrm{(abiotic)}}}$ and $Q_{\mathrm{ADS}_{\mathrm{(biotic)}}}$ are the surface charge density for the abiotic and biotic media, respectively.

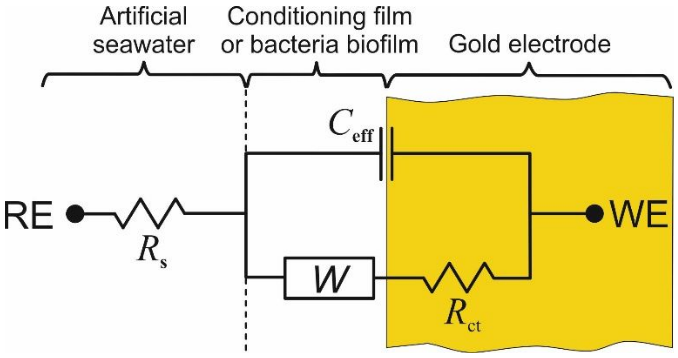

Figure S2. The equivalent circuit model for a gold electrode surface in an artificial seawater medium. R_s represents the solution resistance, R_{ct} the charge transfer resistance, C_{eff} effective capacitance and W the Warburg impedance.

Impedance components such as resistive and diffusion where charge is transferred across the interface, are classified as faradaic processes. A capacitance component will correspond to charging effects. For a biofilmed surface, the overall capacitive response will be a combination of the EPS matrix capacitance inherent to the biofilm, the capacitance of the conditioning layer, and also the double layer capacitance at the gold surface.

(a)

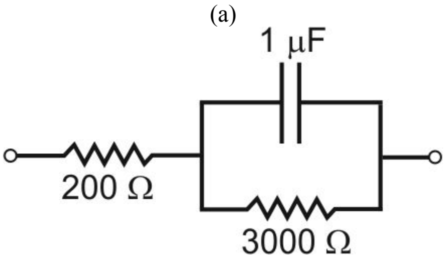

(b)

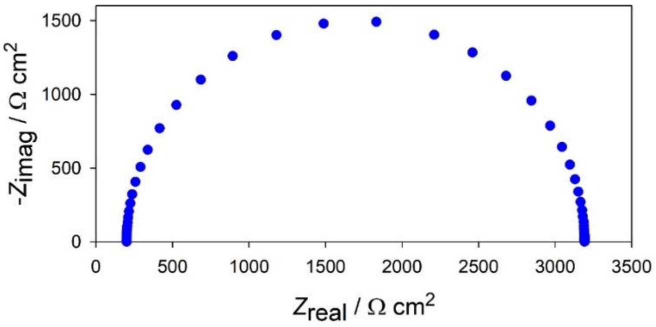

(c)

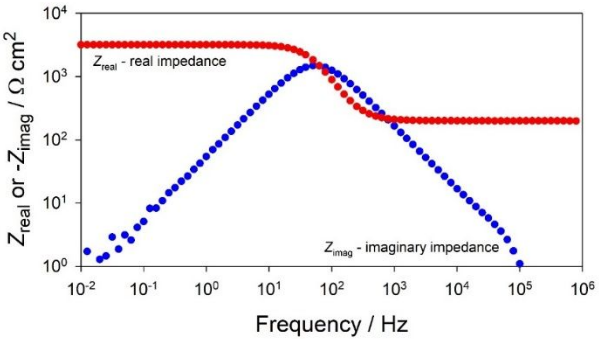

Figure S3. **(a)** a simple Randles equivalent circuit; **(b)** the resulting Nyquist plot and **(c)** the two Bode plots: Z_{real} *vs. f* and $-Z_{imag}$ *vs. f*.

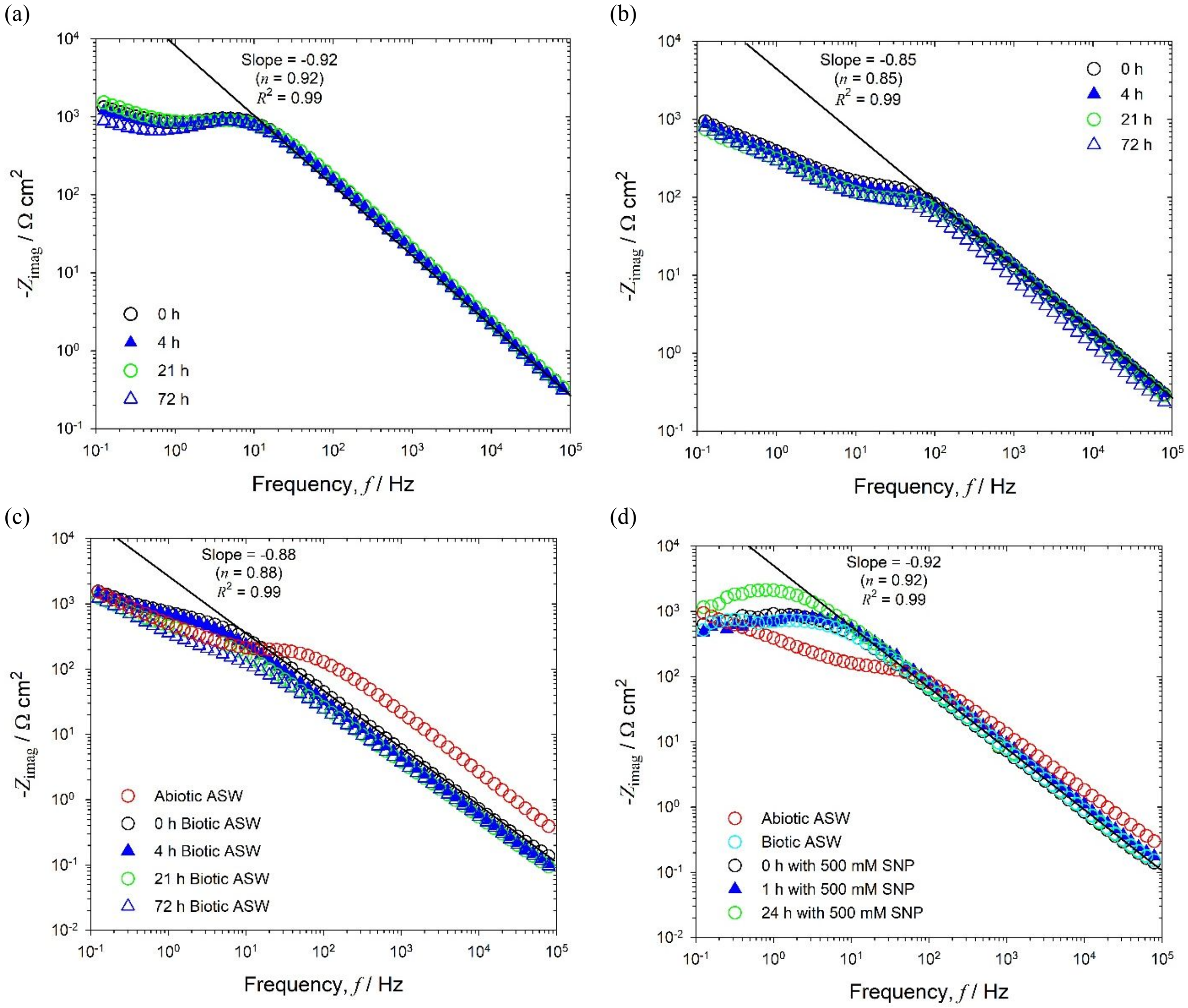

Figure S4. Bode imaginary impedance ($-Z_{imag}$ *vs.*) plots for **(a)** abiotic 3.5 wt.% NaCl test medium (#1), **(b)** abiotic artificial seawater test medium (#2), **(c)** biotic artificial seawater test medium (#3) and **(d)** biotic artificial seawater (72 h) and exogenous NO exposure – test medium #4.

The n coefficients using this graphical method are presented alongside other evaluated EIS data in Supplementary Information Table S2.

Confocal microscopy. BacLight combines a green fluorescent SYTO9 stain, which penetrates intact cells (which can either be alive or dead) and a red fluorescent propidium iodide (PI) stain for dead or dying cells with damaged membranes only. Using the Leica TCS SP2, a series of confocal image stacks were collected at 0.5 μm intervals along the *z*-direction. Image deconvolution was carried out utilizing the Leica Confocal Software (LCS) version 2.61 and the biofilm stacks were subsequently projected to a view plane to assess biofilm thickness. ImageJ software was used for percentage coverage analyses on the gold surface images to ultimately assess the numbers of adhered bacteria cells utilizing a relevant size criterion for one adhered *Pseudoalteromonas* sp. cell (length between 2 μm – 3 μm and a diameter of 0.5 μm^{22}). Suitable thresholds were applied to the Leica TCS SP2 images, thus rendering biofilm cell clusters in black and the corresponding channels in white.[23]

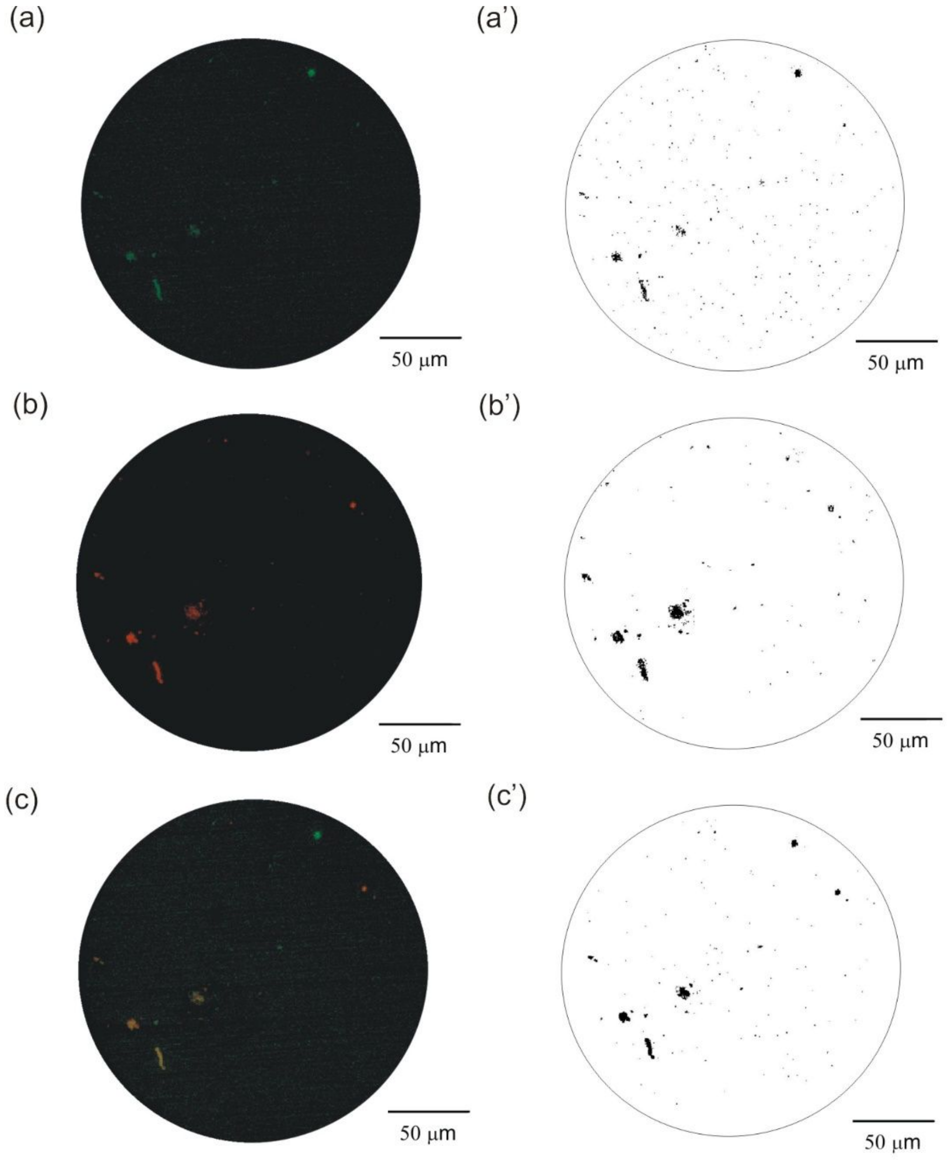

Figure S5. Confocal microscopy of a gold electrode stained with the *Bac*Light™ viability kit after a 72 h immersion in abiotic 3.5 wt.% NaCl: **(a)** Live, **(b)** Dead and **(c)** Live/Dead with the corresponding binary (black and white) images **(a')**, **(b')** and **(c')** using ImageJ.

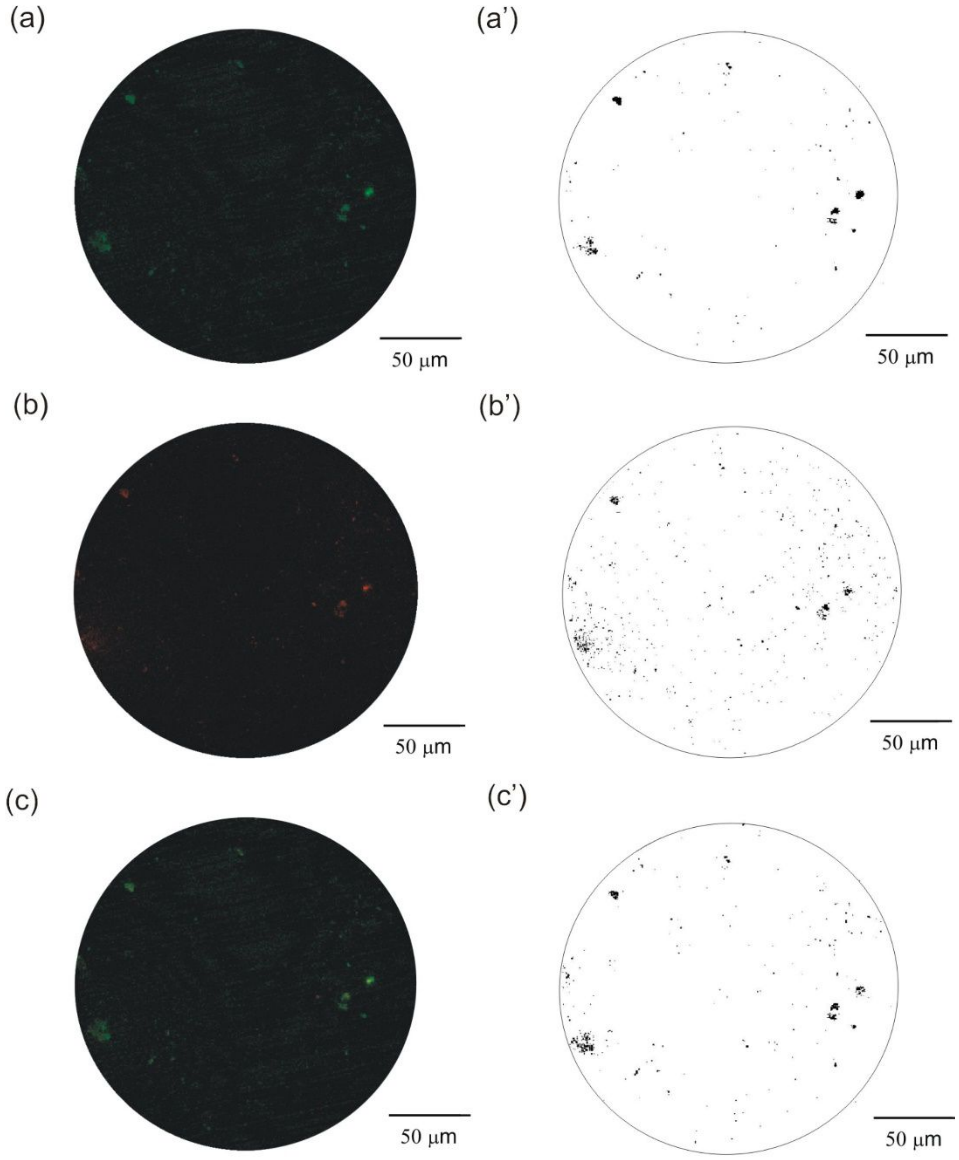

Figure S6. Confocal microscopy of a gold electrode stained with the *Bac*Light^{TM} viability kit after a 72 h immersion in abiotic ASW: **(a)** Live, **(b)** Dead and **(c)** Live/Dead with the corresponding binary (black and white) images **(a')**, **(b')** and **(c')** using ImageJ.

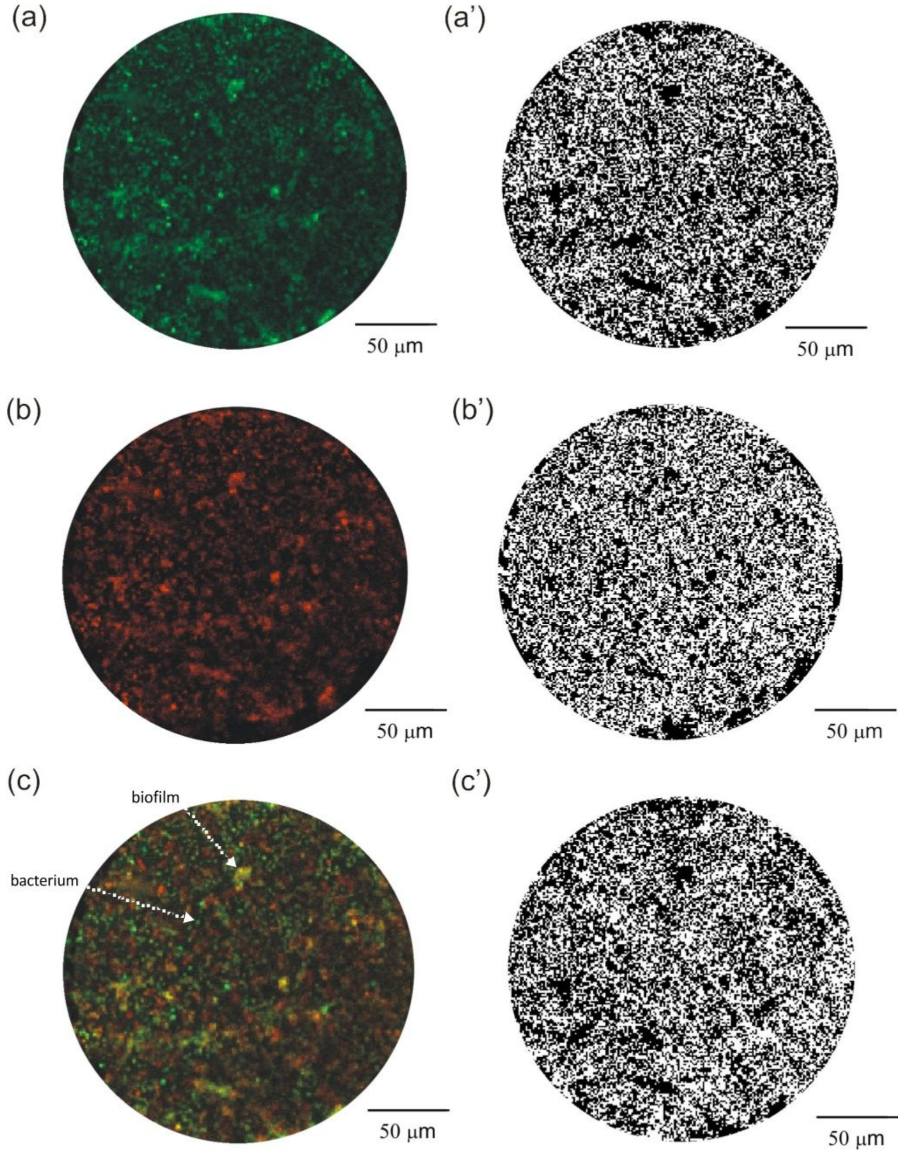

Figure S7. Confocal microscopy of a gold electrode stained with the *Bac*Light^{TM} viability kit after a 72 h immersion in biotic ASW: **(a)** Live, **(b)** Dead and **(c)** Live/Dead with the corresponding binary (black and white) images **(a')**, **(b')** and **(c')** using ImageJ.

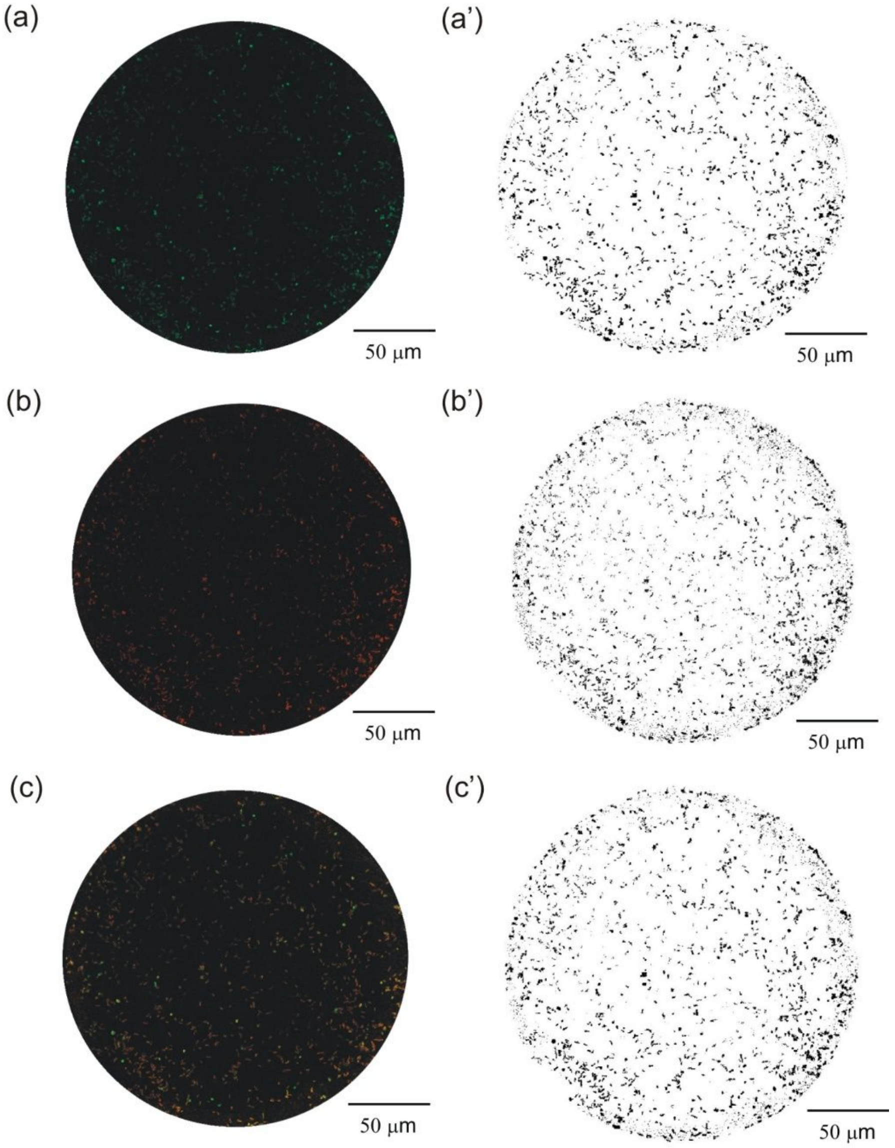

Figure S8. Confocal microscopy of a gold 72 h-old biofilmed electrode stained with the BacLightTM viability kit after a 24 h immersion using 500 nM of the NO donor SNP: **(a)** Live, **(b)** Dead and **(c)** Live/Dead with the corresponding binary (black and white) images **(a')**, **(b')** and **(c')** using ImageJ.

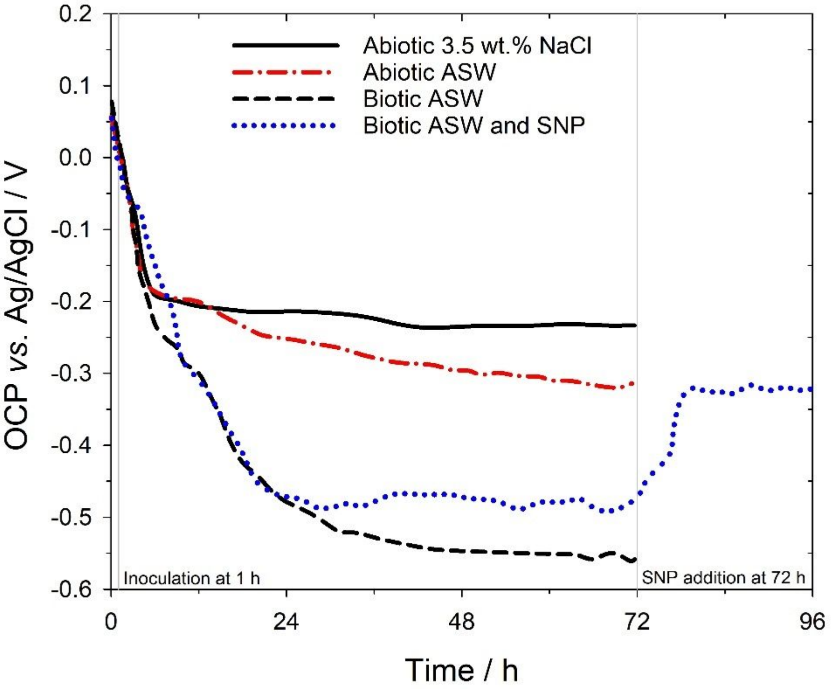

Figure S9. Open-circuit potential comparison for the aerobic 3.5 wt.% NaCl solution, abiotic and biotic ASW media over 72 h, and subsequent 24 h exogenous exposure to the NO donor SNP in the biotic ASW medium.

Open-circuit potentials. Supplementary Information Figure S9 shows the OCP profiles over 72 h for the 3.5 wt.% NaCl, abiotic ASW and biotic ASW test media. Overall, the OCPs shift electronegatively towards the ORR region, between about +0.100 and –0.225 V (*vs.* Ag/AgCl) for the NaCl test solution, and –0.325 V (*vs.* Ag/AgCl) for the abiotic ASW, and –0.550 V (*vs.* Ag/AgCl) for the biotic ASW. Whereas the OCPs gradually stabilize within the first hours for the NaCl and abiotic ASW solutions, those for the biotic conditions exhibit a more pronounced shift towards more electronegative potentials. This is attributable to the development of an interfacial charge distribution when achieving a steady-state condition, *i.e.*, the double layer for the NaCl solution where chloride ions can negatively charge the gold surface and yield chloro-complexes (under low flowing conditions, thus affecting the gold interface), and electrode polarization due to the presence of adsorbed organic material (the conditioning film) associated with an overall change in the adsorbed organic species and the kinetics of the ORR for the abiotic ASW.[24, 25] For the biotic ASW, the polarization process of the gold surface due to the biofilm formation is consistent with.[26, 27]

In particular, the negative potential drift in Supplementary Information Figure S9 reflects an increased surface heterogeneity in the presence of an adsorption organic film or biofilm (for the abiotic and biotic ASW), thereby the exposed gold active surface area decreases and can subsequently influence the ORR.[28] Similarly, the exact interfacial mechanism may be more complex with a possible catalase influence that shifts the OCPs electronegatively.[29] After NO donor addition to the biotic ASW, the OCP increased electropositively towards a potential plateau at about –0.300 V from a baseline of –0.475 V. This plateau corresponds to potentials measured in abiotic ASW, thus indicating the gold electrode was depolarized associated with biofilm dispersal. Although the EIS data have been obtained for different OCPs, this work has outlined the naturally occurring bacterial biofilm initiation and propagation on a gold surface (without applied potentials). It is well-documented that NO is electrochemically active; depending on the electrode type and solution pH, it can undergo a one electron reduction at potentials ranging between −0.500 V and −1.400 V (*vs.* Ag/AgCl) to form a nitrosyl anion (NO^-) which is highly unstable in aqueous environments.[30, 31] The electrochemical detection of NO within this potential range is subject to severe interference of dissolved oxygen whose electroreduction is thermodynamically more favorable than that of NO. Similarly, NO reacts with reactive oxygen intermediates to form peroxynitriate ($ONOO^-$), which is also a potent antimicrobial species.[32]

Table S1. Biochemical characteristics of the test media[33]

Test media	Biochemical characteristics	Experimental details
**1.** 3.5 wt.% NaCl, pH 7.9, conductivity: 50.4 mS cm^{-1}	Sterile – **Abiotic**	Baseline - **Abiotic**
**2.** 0.1 % (w/v) tryptone and 0.07 % (w/v) yeast extract in ASW, pH 8.1, conductivity: 49.5 mS cm^{-1}	Organic matter – Sterile – **Abiotic**	Baseline for conditioning film (adsorbed organic layer) – **Abiotic ASW**
**3.** 0.1 % (w/v) tryptone and 0.07 % (w/v) yeast extract in ASW with 200 µL aliquot of 2 h *Pseudoalteromonas* sp. culture, pH 8.1, conductivity: 49.9 mS cm^{-1}	Organic matter – Non-sterile – **Biotic**	Biofilm growth – **Biotic ASW**
**4.** 500 nM of the nitric oxide donor SNP in 0.1 % (w/v) tryptone and 0.07 % (w/v) yeast extract in ASW, pH 8.1 on a 72 h biofilmed surface, conductivity: 47.9 mS cm^{-1}	Organic matter – Non-sterile – **Biotic** – with a nitric oxide donor addition after 72 h	Biofilm dispersal – **500 nM SNP in ASW**

Table S2. Evaluated EIS data for the 0.2 mm diameter gold electrode and various test media. Where the exponent parameter n was determined from the $-Z_{imag}$ *vs. f* plots and the number of *Pseudoalteromonas* cells assessed using Eqn. S5

**Test media**		Time / h	n	Y_o / $\mu\Omega^{-1}$ cm^{-2} s^n	R_{ct} / Ω cm^2	C_{eff} / µF cm^{-2}	Q_{ads} / µC cm^{-2}	Number of cells × 10^6 / cells cm^{-2}
#1	**Abiotic NaCl**	0	0.92	14.5 ± 0.3	1605 ± 83	10.9 ± 0.1	N/A	N/A
	–	4	0.92	14.8 ± 0.3	1579 ± 95	11.2 ± 0.1	N/A	N/A
	–	21	0.92	14.1 ± 0.3	1508 ± 71	10.6 ± 0.1	N/A	N/A
	–	72	0.92	14.0 ± 0.4	1505 ± 89	10.5 ± 0.2	N/A	N/A
#2	**Abiotic ASW**	0	0.85	28.8 ± 0.9	220 ± 8	11.8 ± 0.1	4.7 ± 0.1	N/A
	–	4	0.85	26.2 ± 0.9	180 ± 8	10.2 ± 0.1	4.1 ± 0.1	N/A
	–	21	0.85	28.1 ± 0.9	161 ± 6	10.8 ± 0.1	4.4 ± 0.1	N/A
	–	72	0.83	43.7 ± 1.5	172 ± 6	16.0 ± 0.2	6.5 ± 0.1	N/A
#3	**Abiotic ASW**	–1	0.89	20.1 ± 0.4	331 ± 13	10.8 ± 0.1	4.3 ± 0.1	N/A
	**Biotic ASW**	0	0.88	76.2 ± 1.6	950 ± 60	53.3 ± 0.5	21.4 ± 0.2	17.1 ± 0.1
	–	4	0.87	103.2 ± 1.5	876 ± 45	72.1 ± 0.5	28.9 ± 0.1	24.6 ± 0.1
	–	21	0.85	142.8 ± 2.1	618 ± 31	93.0 ± 0.6	37.4 ± 0.2	33.1 ± 0.1
	–	72	0.85	167.6 ± 2.8	296 ± 626	98.6 ± 0.9	39.7 ± 0.4	35.3 ± 0.3
#4	**Abiotic ASW**	–1	0.85	31.1 ± 0.9	233 ± 9	13.0 ± 0.5	5.2 ± 0.1	N/A
	**Biotic ASW**	72	0.92	95.3 ± 0.8	759 ± 66	75.8 ± 1.3	30.4 ± 0.1	25.3 ± 0.1
	**500 nM SNP in ASW**	0	0.92	55.1 ± 0.6	886 ± 57	42.4 ± 0.7	17.0 ± 0.1	11.8 ± 0.1
		1	0.93	37.9 ± 0.5	946 ± 60	29.5 ± 0.6	11.8 ± 0.1	6.6 ± 0.1
		24	0.93	32.2 ± 0.4	3251 ± 150	27.2 ± 0.5	10.9 ± 0.1	5.7 ± 0.1

Table S3. EIS data for the gold/3.5 wt.% NaCl interface

Conditioning		OCP / V (*vs.* Ag/AgCl)	R_s / Ω cm^2	R_{ct} / Ω cm^2	$Z_{C_{dl}}$			C_{eff} (C_{dl})	Z_W		χ^2 / 10^{-4}
					Y_o / μΩ^{-1} cm^{-2} s^n	n	R^2	μF cm^{-2}	Y_o / μΩ^{-1} cm^{-2} s^n	n	
**3.5 % NaCl**	**0 h**	+0.090	0.165 ± 0.006	1605 ± 83	14.5 ± 0.3	0.92	0.99	10.9 ± 0.1	517 ± 21	0.38	1.6
	**4 h**	–0.070	0.163 ± 0.006	1579 ± 95	14.8 ± 0.3	0.92	0.99	11.2 ± 0.1	541 ± 25	0.36	2.1
	**21 h**	–0.215	0.167 ± 0.006	1508 ± 71	14.1 ± 0.3	0.92	0.99	10.6 ± 0.1	464 ± 15	0.41	1.7
	**72 h**	–0.225	0.164 ± 0.006	1505 ± 89	14.0 ± 0.4	0.92	0.99	10.5 ± 0.2	475 ± 17	0.40	2.9

Table S4. EIS data for the gold/abiotic ASW interface

Conditioning		OCP / V (*vs.* Ag/AgCl)	R_s / Ω cm^2	R_{ct} / Ω cm^2	$Z_{C_{con}}$			C_{eff} (C_{con})	Q_{ads}	Z_W		χ^2 / 10^{-4}
					Y_o / μΩ^{-1} cm^{-2} s^n	n	R^2	μF cm^{-2}	μC cm^{-2}	Y_o / μΩ^{-1} cm^{-2} s^n	n	
**Abiotic ASW**	**0 h**	+0.085	0.167 ± 0.008	220 ± 8	28.8 ± 0.9	0.85	0.99	11.8 ± 0.1	4.7	792 ± 9	0.49	7.6
	**4 h**	–0.070	0.169 ± 0.006	180 ± 8	26.2 ± 0.9	0.85	0.99	10.2 ± 0.1	4.1	837 ± 10	0.49	8.1
	**21 h**	–0.250	0.170 ± 0.005	161 ± 6	28.1 ± 0.9	0.85	0.99	10.8 ± 0.1	4.4	987 ± 12	0.50	10.5
	**72 h**	–0.325	0.166 ± 0.008	172 ± 6	43.7 ± 1.5	0.83	0.99	16.0 ± 0.2	6.5	1020 ± 13	0.52	14.8

Table S5. EIS data for the gold/biotic ASW interface (abiotic: –1 h before inoculation)

Conditioning		OCP / V (*vs.* Ag/AgCl)	R_s / Ω cm^2	R_{ct} / Ω cm^2	$Z_{C_{con}}$			C_{eff} (C_{con})	$Z_{C_{EPS}}$			C_{eff} (C_{EPS})	Q_{ads}	Number of cells	Z_W		χ^2 / 10^{-4}
					Y_o / μΩ^{-1} cm^{-2} s^n	n	R^2	μF cm^{-2}	Y_o / μΩ^{-1} cm^{-2} s^n	n	R^2	μF cm^{-2}	μC cm^{-2}	cells cm^{-2} × 10^6	Y_o / μΩ^{-1} cm^{-2} s^n	n	
Abiotic ASW	–1 h	+0.090	0.163 ± 0.007	331 ± 13	20.1 ± 0.4	0.89	0.99	10.8 ± 0.1	N/A	N/A	N/A	N/A	4.3	N/A	567 ± 20	0.53	3.1
Biotic ASW	0 h	+0.085	0.170 ± 0.004	950 ± 60	N/A	N/A	N/A	N/A	76.2 ± 1.6	0.88	0.99	53.3 ± 0.5	21.4	17.1	582 ± 15	0.54	4.9
	4 h	–0.075	0.168 ± 0.003	876 ± 45	N/A	N/A	N/A	N/A	103.2 ± 1.5	0.87	0.99	72.1 ± 0.5	28.9	24.6	636 ± 11	0.55	4.8
	21 h	–0.460	0.165 ± 0.003	618 ± 31	N/A	N/A	N/A	N/A	142.8 ± 2.1	0.85	0.99	93.0 ± 0.6	37.4	33.1	707 ± 13	0.56	5.4
	72 h	–0.560	0.169 ± 0.003	296 ± 26	N/A	N/A	N/A	N/A	167.6 ± 2.8	0.85	0.99	98.6 ± 0.9	39.7	35.3	680 ± 11	0.59	4.1

Table S6. EIS data for the gold/biotic ASW interface after treatment with 500 nM of NO donor SNP (abiotic: –1 h before inoculation and biotic growth for 72 h)

Conditioning		OCP / V (*vs.* Ag/AgCl)	R_s / Ω cm^2	R_{ct} / Ω cm^2	$Z_{C_{con}}$			C_{eff} (C_{con})	$Z_{C_{EPS}}$			C_{eff} (C_{EPS})	Q_{ads}	Number of cells	Z_W		χ^2 / 10^{-4}
					Y_o / μΩ^{-1} cm^{-2} s^n	n	R^2	μF cm^{-2}	Y_o / μΩ^{-1} cm^{-2} s^n	n	R^2	μF cm^{-2}	μC cm^{-2}	cells cm^{-2} × 10^6	Y_o / μΩ^{-1} cm^{-2} s^n	n	
Abiotic ASW	–1 h	+0.080	0.162 ± 0.006	233 ± 9	31.1 ± 0.9	0.85	0.99	13.0 ± 0.5	N/A	N/A	N/A	N/A	5.2	N/A	795 ± 10	0.49	6.5
Biotic ASW	72 h	–0.470	0.164 ± 0.003	759 ± 66	N/A	N/A	N/A	N/A	95.3 ± 0.8	0.92	0.99	75.8 ± 1.3	30.5	25.3	662 ± 78	0.24	12.5
500 nM SNP in ASW	0 h	–0.470	0.160 ± 0.003	886 ± 57	N/A	N/A	N/A	N/A	55.1 ± 0.6	0.92	0.99	42.4 ± 0.7	17.0	11.8	564 ± 10	0.24	11.5
	1 h	–0.470	0.169 ± 0.004	946 ± 60	N/A	N/A	N/A	N/A	37.9 ± 0.5	0.93	0.99	29.5 ± 0.6	11.9	6.6	636 ± 11	0.19	8.1
	24 h	–0.325	0.168 ± 0.004	3251 ± 150	N/A	N/A	N/A	N/A	32.2 ± 0.4	0.93	0.99	27.2 ± 0.5	10.9	5.7	427 ± 13	0.19	15.2

REFERENCES

(1) Jackson, D. R.; Omanovic, S.; Roscoe, S. G. Electrochemical Studies of the Adsorption Behavior of Serum Proteins on Titanium. *Langmuir* **2000**, *16* (12), 5449-5457. DOI: 10.1021/la991497x.
(2) Moulton, S. E.; Barisci, J. N.; Bath, A.; Stella, R.; Wallace, G. G. Studies of double layer capacitance and electron transfer at a gold electrode exposed to protein solutions. *Electrochimica Acta* **2004**, *49* (24), 4223-4230. DOI: https://doi.org/10.1016/j.electacta.2004.03.034.
(3) Omanovic, S.; Roscoe, S. G. Electrochemical Studies of the Adsorption Behavior of Bovine Serum Albumin on Stainless Steel. *Langmuir* **1999**, *15* (23), 8315-8321. DOI: 10.1021/la990474f.
(4) Smiechowski, M. F.; Lvovich, V. F.; Roy, S.; Fleischman, A.; Fissell, W. H.; Riga, A. T. Electrochemical detection and characterization of proteins. *Biosensors and Bioelectronics* **2006**, *22* (5), 670-677. DOI: https://doi.org/10.1016/j.bios.2006.02.008.
(5) Wright, J. E. I.; Cosman, N. P.; Fatih, K.; Omanovic, S.; Roscoe, S. G. Electrochemical impedance spectroscopy and quartz crystal nanobalance (EQCN) studies of insulin adsorption on Pt. *Journal of Electroanalytical Chemistry* **2004**, *564*, 185-197. DOI: https://doi.org/10.1016/j.jelechem.2003.10.031.
(6) Brug, G. J.; van den Eeden, A. L. G.; Sluyters-Rehbach, M.; Sluyters, J. H. The analysis of electrode impedances complicated by the presence of a constant phase element. *Journal of Electroanalytical Chemistry and Interfacial Electrochemistry* **1984**, *176* (1), 275-295. DOI: https://doi.org/10.1016/S0022-0728(84)80324-1.
(7) Hirschorn, B.; Orazem, M. E.; Tribollet, B.; Vivier, V.; Frateur, I.; Musiani, M. Determination of effective capacitance and film thickness from constant-phase-element parameters. *Electrochimica Acta* **2010**, *55* (21), 6218-6227. DOI: https://doi.org/10.1016/j.electacta.2009.10.065.
(8) Mulder, W. H.; Sluyters, J. H.; Pajkossy, T.; Nyikos, L. Tafel current at fractal electrodes: Connection with admittance spectra. *Journal of Electroanalytical Chemistry and Interfacial Electrochemistry* **1990**, *285* (1), 103-115. DOI: https://doi.org/10.1016/0022-0728(90)87113-X.
(9) Kim, C.-H.; Pyun, S.-I.; Kim, J.-H. An investigation of the capacitance dispersion on the fractal carbon electrode with edge and basal orientations. *Electrochimica Acta* **2003**, *48* (23), 3455-3463. DOI: https://doi.org/10.1016/S0013-4686(03)00464-X.
(10) Schiller, C. A.; Strunz, W. The evaluation of experimental dielectric data of barrier coatings by means of different models. *Electrochimica Acta* **2001**, *46* (24), 3619-3625. DOI: https://doi.org/10.1016/S0013-4686(01)00644-2.
(11) Jorcin, J.-B.; Orazem, M. E.; Pébère, N.; Tribollet, B. CPE analysis by local electrochemical impedance spectroscopy. *Electrochimica Acta* **2006**, *51* (8), 1473-1479. DOI: https://doi.org/10.1016/j.electacta.2005.02.128.
(12) Oldham, K. B. The RC time “constant” at a disk electrode. *Electrochemistry Communications* **2004**, *6* (2), 210-214. DOI: https://doi.org/10.1016/j.elecom.2003.12.002.
(13) Barsoukov, E.; Macdonald, J. R. Impedance Spectroscopy: Theory, Experiment, and Applications. **2005**.
(14) McAdams, E. T.; Lackermeier, A.; McLaughlin, J. A.; Macken, D.; Jossinet, J. The linear and non-linear electrical properties of the electrode-electrolyte interface. *Biosensors and Bioelectronics* **1995**, *10* (1), 67-74. DOI: https://doi.org/10.1016/0956-5663(95)96795-Z.
(15) Orazem, M. E.; Tribollet, B. *Electrochemical Impedance Spectroscopy*; John Wiley & Sons, 2008. DOI: DOI:10.1002/9780470381588.
(16) Andoralov, V. M.; Tarasevich, M. R.; Tripachev, O. V. Oxygen reduction reaction on polycrystalline gold. Pathways of hydrogen peroxide transformation in the acidic medium. *Russian Journal of Electrochemistry* **2011**, *47* (12), 1327-1336. DOI: https://doi.org/10.1134/S1023193511120020.
(17) Ge, X.; Sumboja, A.; Wuu, D.; An, T.; Li, B.; Goh, F. W. T.; Hor, T. S. A.; Zong, Y.; Liu, Z. Oxygen Reduction in Alkaline Media: From Mechanisms to Recent Advances of Catalysts. *ACS Catalysis* **2015**, *5* (8), 4643-4667. DOI: https://doi.org/10.1021/acscatal.5b00524.
(18) Shao, M. H.; Adzic, R. R. Spectroscopic Identification of the Reaction Intermediates in Oxygen Reduction on Gold in Alkaline Solutions. *The Journal of Physical Chemistry B* **2005**, *109* (35), 16563-16566. DOI: 10.1021/jp053450s.
(19) Jusys, Z.; Behm, R. J. The Effect of Anions and pH on the Activity and Selectivity of an Annealed Polycrystalline Au Film Electrode in the Oxygen Reduction Reaction-Revisited. *ChemPhysChem* **2019**, *20* (24), 3276-3288. DOI: https://doi.org/10.1002/cphc.201900960.
(20) Poortinga, A. T.; Bos, R.; Busscher, H. J. Measurement of charge transfer during bacterial adhesion to an indium tin oxide surface in a parallel plate flow chamber. *Journal of Microbiological Methods* **1999**, *38* (3), 183-189. DOI: https://doi.org/10.1016/S0167-7012(99)00100-1.
(21) van der Wal, A.; Norde, W.; Zehnder, A. J. B.; Lyklema, J. Determination of the total charge in the cell walls of Gram-positive bacteria. *Colloids and Surfaces B: Biointerfaces* **1997**, *9* (1), 81-100. DOI: https://doi.org/10.1016/S0927-7765(96)01340-9.
(22) Yuan, S. J.; Pehkonen, S. O. Microbiologically influenced corrosion of 304 stainless steel by aerobic Pseudomonas NCIMB 2021 bacteria: AFM and XPS study. *Colloids and Surfaces B: Biointerfaces* **2007**, *59* (1), 87-99. DOI: https://doi.org/10.1016/j.colsurfb.2007.04.020.
(23) Stoodley, P.; Yang, S.; Lappin-Scott, H.; Lewandowski, Z. Relationship between mass transfer coefficient and liquid flow velocity in heterogenous biofilms using microelectrodes and confocal microscopy. *Biotechnology and Bioengineering* **1997**, *56* (6), 681-688. DOI: https://doi.org/10.1002/(SICI)1097-0290(19971220)56:6<681::AID-BIT11>3.0.CO;2-B.
(24) Donlan, R. M. Biofilms: Microbial Life on Surfaces. *Emerg. Infect. Dis* **2002**, *8* (9), 881-890. DOI: https://doi.org/10.3201/eid0809.020063.

(25) Franks, W.; Schenker, I.; Schmutz, P.; Hierlemann, A. Impedance characterization and modeling of electrodes for biomedical applications. *IEEE Transactions on Biomedical Engineering* **2005**, *52* (7), 1295-1302. DOI: 10.1109/TBME.2005.847523.
(26) Nercessian, D.; Duville, F. B.; Desimone, M.; Simison, S.; Busalmen, J. P. Metabolic turnover and catalase activity of biofilms of Pseudomonas fluorescens (ATCC 17552) as related to copper corrosion. *Water Research* **2010**, *44* (8), 2592-2600. DOI: https://doi.org/10.1016/j.watres.2010.01.014.
(27) Busalmen, J. P.; de Sánchez, S. R. Changes in the electrochemical interface as a result of the growth of Pseudomonas fluorescens biofilms on gold. *Biotechnology and Bioengineering* **2003**, *82* (5), 619-624. DOI: https://doi.org/10.1002/bit.10600.
(28) Bard, A. J.; Faulkner, L. R. *Electrochemical Methods: Fundamentals and Applications*; Wiley, 2000.
(29) Washizu, N.; Katada, Y.; Kodama, T. Role of H_2O_2 in microbially influenced ennoblement of open circuit potentials for type 316L stainless steel in seawater. *Corrosion Science* **2004**, *46* (5), 1291-1300. DOI: https://doi.org/10.1016/j.corsci.2003.09.018.
(30) Bedioui, F.; Griveau, S. Electrochemical Detection of Nitric Oxide: Assessement of Twenty Years of Strategies. *Electroanalysis* **2013**, *25* (3), 587-600. DOI: https://doi.org/10.1002/elan.201200306.
(31) Ciszewski, A.; Milczarek, G. Electrochemical detection of nitric oxide using polymer modified electrodes. *Talanta* **2003**, *61* (1), 11-26. DOI: https://doi.org/10.1016/S0039-9140(03)00355-2.
(32) Barraud, N.; Hassett, D. J.; Hwang, S.-H.; Rice, S. A.; Kjelleberg, S.; Webb, J. S. Involvement of Nitric Oxide in Biofilm Dispersal of *Pseudomonas aeruginosa*. *Journal of Bacteriology* **2006**, *188* (21), 7344. DOI: 10.1128/JB.00779-06.
(33) Werwinski, S.; Wharton, J. A.; Nie, M.; Stokes, K. R. Electrochemical Sensing and Characterization of Aerobic Marine Bacterial Biofilms on Gold Electrode Surfaces. *ACS Applied Materials & Interfaces* **2021**, *13* (27), 31393-31405. DOI: https://doi.org/10.1021/acsami.1c02669.
